# The Adiponectin‐PP2A Pathway Confers Cognitive Benefits of Physical Exercise Against Chronic Stress‐Induced Tau Hyperphosphorylation in the Hippocampus

**DOI:** 10.1111/acel.70447

**Published:** 2026-03-17

**Authors:** Hui‐Hui Guo, Hai‐Ning Ou, Jia‐Sui Yu, Zi‐Rui Luo, Suk‐Yu Yau, Hector Wing‐Hong Tsang

**Affiliations:** ^1^ Department of Rehabilitation Medicine The Fifth Affiliated Hospital of Guangzhou Medical University Guangzhou China; ^2^ Department of Rehabilitation Medicine Shaoxing People's Hospital Shaoxing China; ^3^ Department of Rehabilitation The 2nd Affiliated Hospital, Guangzhou University of Chinese Medicine Guangzhou China; ^4^ The Second Institute of Clinical Medicine Guangzhou University of Chinese Medicine Guangzhou China; ^5^ Department of Rehabilitation Sciences The Hong Kong Polytechnic University Hong Kong SAR China; ^6^ Mental Health Research Center The Hong Kong Polytechnic University Hong Kong SAR China; ^7^ Key Laboratory of Biological Targeting Diagnosis, Therapy and Rehabilitation of Guangdong Higher Education Institutes The Fifth Affiliated Hospital of Guangzhou Medical University Guangzhou China

**Keywords:** adiponectin, chronic unpredictable stress, hippocampus, physical exercise, protein phosphatase 2A, tau phosphorylation

## Abstract

Protein phosphatase 2A (PP2A) regulates Tau hyperphosphorylation in Alzheimer's disease (AD). This study hypothesized that exercise increases adiponectin levels, activating PP2A to reduce Tau hyperphosphorylation and enhance hippocampal plasticity. The study utilized adiponectin knockout (*Adipo* ^−/−^) and hippocampal‐specific PP2A knockdown (PP2A‐KD) in mice with 3‐week voluntary running and/or chronic stress to assess changes in Tau phosphorylation, adult neurogenesis, and cognitive performance. Running improved cognitive deficits and reduced Tau hyperphosphorylation in association with increased adiponectin levels and enhanced PP2A activity in stressed mice. *A*diponectin deficiency impaired cognitive performance, increased Tau phosphorylation, and decreased PP2A activity. Mechanistically, adiponectin is dispensable for running to increase PP2A activity, reduce Tau hyperphosphorylation, and restore hippocampal neurogenesis, leading to cognitive improvement. Hippocampal‐specific PP2A knockdown diminished the beneficial effects of running, indicating that PP2A is downstream of adiponectin's action. This study provides mechanistic insights into how exercise reduces AD‐like neuropathology, emphasizing the critical role of the adiponectin‐PP2A pathway in mitigating Tau hyperphosphorylation and suggesting a potential therapeutic target for AD through modulation of this pathway.

## Graphical abstract

Physical exercise ameliorates cognitive deficits and attenuates Tau hyperphosphorylation under chronic stress by elevating circulating adiponectin levels. Upon receptor binding, adiponectin modulates the LCMT1/PME‐1 regulatory axis to promote PP2A methylation and catalytic function. This cascade reduces pathological Tau phosphorylation and stabilizes microtubules, ultimately fostering adult hippocampal neurogenesis and restoring learning and memory under chronic stress.

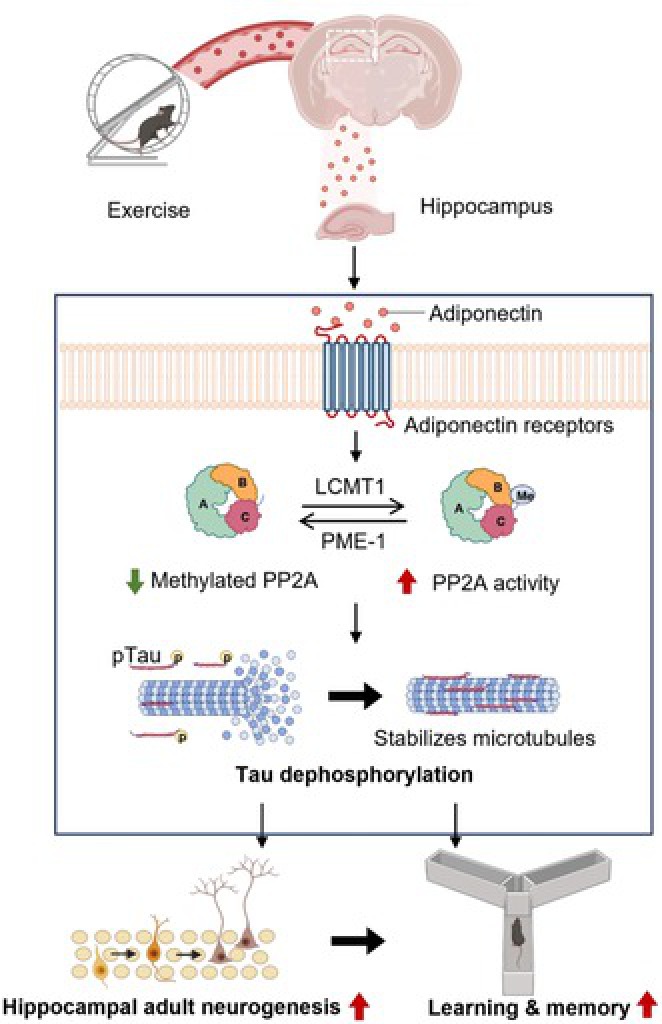

**Abbreviations**
AD: Alzheimer's disease
AdipoRs: AdipoR1 and AdipoR2
AKT: protein kinase B
CUS: chronic unpredictable stress
CUSE: chronic unpredictable stress with exercise
DCX: doublecortin
ELISA: enzyme‐linked immunosorbent assay
EPM: elevated plus maze
FST: forced swim test
GSK‐3β: glycogen synthase kinase‐3β
KD: knock down
LCMT1: leucine carboxyl methyltransferase 1
NOR: novel object recognition
OFT: open field test
PBS: phosphate‐buffered saline
PI3K: phosphatidylinositol‐3‐kinase
PME‐1: phosphatase methylesterase 1
PP2A: protein phosphatase 2A
PPARγ: peroxisome proliferator‐activated receptor gamma
PSD‐95: postsynaptic density protein‐95
WT: wild‐type

## 1 Introduction

Alzheimer's disease (AD) is a progressive neurodegenerative disorder and the leading cause of dementia worldwide (Masters et al. 2015). AD is diagnosed through clinical assessment, cognitive testing, and, when available, biomarkers such as amyloid‐beta and tau levels or neuroimaging (Jack Jr. et al. 2024). Current treatments, including cholinesterase inhibitors and memantine, provide limited symptomatic relief but do not halt disease progression. Although monoclonal antibodies targeting beta‐amyloid have been introduced, their long‐term efficacy is still under evaluation. Therefore, physical exercise as a non‐pharmacological intervention is recognized as an effective way to improve cognitive function in AD patients.

Physical exercise attenuates cognitive decline (Delgado‐Peraza et al. 2023; S. S. Zhang et al. 2022) and alleviates depressive symptoms (Noetel et al. 2024). Mechanistically, exercise enhances hippocampal neuroplasticity, a critical substrate for learning, memory, and emotional regulation (Liao and Losonczy 2024; Pronier et al. 2023). In animal models, physical activity reduces AD‐related Tau pathology (Ohia‐Nwoko et al. 2014), and increases the number of hippocampal neural stem cells (Gao et al. 2020; Pinar et al. 2018), proliferating cells (Leiter et al. 2023; Yau et al. 2014), and immature neurons (Gao et al. 2020; Leiter et al. 2023; Nicolas et al. 2024; Yau et al. 2014). Despite these established benefits, the precise molecular mechanisms by which exercise reduces Tau accumulation and its associated neurotoxicity are still largely unknown.

Hyperphosphorylation of the Tau protein is an early and persistent pathological event in AD (Lantero‐Rodriguez et al. 2024). Excessive Tau phosphorylation disrupts microtubule stability, promotes intracellular aggregation, and impairs synaptic function (Congdon et al. 2023), ultimately contributing to hippocampal dysfunction and memory decline. Protein phosphatase 2A (PP2A) is regarded as the major Tau phosphatase in the human brain (Z. Hu et al. 2023; R. Liu et al. 2008). Inhibition of PP2A activity can lead to Tau hyperphosphorylation, neurodegeneration, and cognitive impairment in AD patients (W. Hu et al. 2022). In addition, PP2A can regulate hippocampal synaptic plasticity (Maltsev and Balaban 2021), proliferation of progenitor cells (Sullivan et al. 2016), and self‐renewal of neural stem cells (C. Wang et al. 2009). Interestingly, recent evidence suggests that exercise enhances PP2A activity (W. Zhang et al. 2021), indicating the possible involvement of PP2A in the neuroprotective effects of physical exercise against Tau pathology.

Chronic stress is increasingly recognized as a risk factor for cognitive decline and AD progression. Both human and animal studies show that prolonged stress exposure impairs learning and memory while promoting depressive‐like behaviors (Jiang et al. 2013; McKlveen et al. 2016; Radford et al. 2017; Szala‐Rycaj et al. 2023). Furthermore, chronic stress promotes Tau protein accumulation in the brain, leading to neurodegeneration (Dioli et al. 2023). Clinically, anxiety can exacerbate the progression of mild cognitive impairment in patients with AD (Becker et al. 2018; Santabárbara et al. 2019). Although exercise is known to reverse stress‐induced impairments in adult hippocampal neurogenesis (Yau et al. 2014), dendritic complexity (Lee et al. 2021), and synaptic plasticity (Lourenco et al. 2019), the molecular mediators linking exercise to reduced Tau pathology remain unclear.

Adiponectin is widely known for its insulin‐sensitizing, anti‐diabetic, anti‐inflammatory, anti‐fibrotic, anti‐apoptotic, and anti‐atherosclerotic properties (Aljafary and Al‐Suhaimi 2022; Kadowaki and Yamauchi 2005; Lihn et al. 2005). Emerging studies have shown that reduction in adiponectin levels could contribute to the development and progression of AD (Letra et al. 2019), increased tau phosphorylation (Ng et al. 2016), and reduced adult hippocampal neurogenesis (D. Zhang et al. 2016). In contrast, adiponectin treatment attenuates Tau hyperphosphorylation induced by intraventricular injection of streptozotocin (Xu et al. 2018). Notably, physical exercise increases adiponectin levels (Formolo et al. 2019; Yau et al. 2014), which contributes to its pro‐neurogenic and pro‐cognitive effects (Yau et al. 2011; E. Zhang et al. 2012). Adiponectin can activate PP2A via the adaptor protein phosphotyrosine interacting with PH domain and leucine zipper1 (APPL1) (Deepa et al. 2011), this led us to speculate that adiponectin may reduce Tau hyperphosphorylation by activating PP2A.

In the present study, we hypothesized that physical exercise increases adiponectin levels to activate PP2A in the hippocampus, thereby reducing Tau phosphorylation and restoring hippocampal neuroplasticity. By delineating this adiponectin–PP2A signaling axis in regulating Tau hyperphosphorylation, our study provides novel mechanistic insight into the neuroprotective effects of physical exercise, and a potential exercise‐mimetic therapeutic strategy for AD, particularly for individuals unable to engage in regular physical exercise.

## 2 Methods and Materials

### 2.1 Animal Models

Adult male WT C57BL/6J mice were purchased from the Central Animal Facilities at PolyU Shenzhen Research Institute. Four‐ to six‐week‐old homozygous (B6;129‐Adipoq^tm1Chan^/J, stock number 008195) adiponectin knockout mice (*Adipo* ^−/−^) were acquired from the Jackson Laboratory. *Adipo* ^−/−^ mice breeding colonies were adopted from previous studies which have confirmed absence of adiponectin expression (Cheng et al. 2025; Yau et al. 2014). Ppp2ca‐flox/wt (Ppp2ca^f/w^) mice (genetic background C57BL/6JGpt, strain ID T018350) were obtained from GemPharmatech Co. Ltd., Guangzhou, China. Ppp2ca^f/f^ mice were bred from Ppp2ca^f/w^ littermate mice to obtain the Ppp2ca‐flox/flox (Ppp2ca^f/f^) mice. Male mice at the age of 4–6 weeks were used for experiments. All the animals were kept in a standard animal holding facility with a 12 h light/dark cycle, temperature (20°C ± 2°C), humidity (40%–60%), and were provided with food and water ad libitum living environment.

### 2.2 Chronic Unpredictable Stress (CUS)

The CUS mouse model was adopted as previously described (Cheng et al. 2025). In brief, the mice were randomly subjected to different stressors at different times of the day continuously for 21 consecutive days. The control mice were housed under the same conditions without exposure to stressors; see the details in Table S1.

### 2.3 Voluntary Wheel Running

Voluntary wheel running was performed as previously described (Yau et al. 2011). Briefly, after an adaptation period of 3–7 days in the holding cages, the mice were given unrestricted access to running wheels in their home cages for a continuous period of 21 days. Two voluntary running wheels (17 cm diameter) were introduced to the cage for 4 weeks and detected by magnetic sensors (CKC TINNER) every day. The control mice (non‐runners) were housed in identical home cages without running wheels. The daily counts of the running wheels revolutions for WT, *Adipo* ^−/−^ and PP2A‐KD mice with voluntary running were monitored and recorded (Figure S1A). Total wheel revolutions were converted to running distance and normalized by the number of mice housed in each cage to estimate the average daily running distance per mouse.

### 2.4 Behavioral Tests

#### 2.4.1 Y‐Maze Task

Spatial memory was assessed via the Y‐maze task as previously described (Yau et al. 2014). In the pretraining phase, the mice were allowed to freely explore the starting arm and familiar arm (30 cm × 6 cm × 8 cm) in the Y‐maze for 5 min. After a four‐hour interval in the home cage, the mice were reintroduced to the maze with the novel arm unblocked and allowed to explore for 5 min. The time spent in each arm and the number of visits to each arm were recorded and analyzed with Ethovision XT15 software (Noldus, Wageningen, The Netherlands). The percentage (%) of time or number of visits was calculated using the following formula: % time/visit = (exploration time or entries in the novel arm/total time or number of entries) × 100.

#### 2.4.2 Novel Object Recognition Test

Working memory performance was assessed using the novel object recognition (NOR) test as previously performed (Yau et al. 2014; W. Zhang et al. 2021). The mice were first allowed to habituate in the open field (50 cm × 50 cm × 50 cm) for 15 min on Day 1. On Day 2, the mice were allowed to explore two identical objects for 10 min. Following a two‐hour interval, the mice were presented with a novel object (N) and a familiar object (F) for 5 min, and the time spent exploring each object was recorded. The exploration index was calculated as N/(*N* + F).

#### 2.4.3 Elevated Plus Maze Test

Anxiety‐like behavior was assessed using the elevated plus maze (EPM) test (S. Li et al. 2021). The mice were placed in a maze elevated 50 cm above the floor, consisting of two open arms (30 cm × 6 cm) and two closed arms (30 cm × 7 cm × 8 cm), and allowed to explore the maze for 5 min. The time spent in each arm and the number of entries into each arm were recorded and analyzed with Noldus Ethovision XT15 software (Noldus, Wageningen, The Netherlands).

#### 2.4.4 Forced Swim Test

Depression‐like behavior was assessed using the forced swim test (FST) according to the established protocols (Yau et al. 2014). The mice were placed into a cylinder measuring 30 cm in height and 15 cm in diameter which was filled with water (23°C ± 2°C). The mice were recorded for 6 min with a camera, and their immobility time was measured. The immobility time during the last 4 min of the test was analyzed in a sample‐blinded manner.

#### 2.4.5 Open Field Test

The open field test (OFT) was used to assess anxiety‐like behavior and locomotor activity. The mice were allowed to explore a clean open field (50 cm × 50 cm × 50 cm) for 10 min in a dimly lit environment, as previously described (Yau et al. 2014). The Ethovision XT15 software (Noldus, Wageningen, The Netherlands) was used to analyze the total distance traveled, mean velocity, time spent, and frequency in the central area during the first 5 min of the test.

### 2.5 Intrahippocampal Injection for Ppp2ca Knockdown

To specifically knockdown hippocampal Ppp2ca gene expression, bilateral intrahippocampal injections targeting the dorsal CA1 subregion (−2.1 mm anterior–posterior, ±1.5 mm medial‐lateral, 1.7 mm dorsal‐ventral) were performed with a stereotaxic apparatus (RWD, Shenzhen, China), Hamilton syringe and needle as previously described (W. Zhang et al. 2021). Both Ppp2ca^f/f^ and Ppp2ca^f/w^ (control) male mice, which carry loxP sites flanking exon(s) of the Ppp2ca gene allowing for Cre‐mediated recombination, were injected with AAV2/9‐CAG‐Cre‐GFP (Brain VTA, China) at a concentration of 1 × 10^12^ (500 nL per hemisphere). The CAG promoter is a synthetic, ubiquitous promoter that drives robust Cre expression in both neuronal and non‐neuronal cell types. Control animals received AAV2/9‐CAG‐GFP without Cre. Injection was performed at a controlled rate of 50 nL/min by using a micro‐syringe pump (World Precision Instrument, USA). The needle was maintained in place for 10 min before retraction. The mice were given a 3‐week recovery period before subsequent experiments were conducted.

### 2.6 Tail Vein Injection for Systemic Adiponectin Overexpression

The murine adiponectin coding sequence (NM_009605.5) was cloned into an AAV transfer plasmid under the control of the CAG promoter with a porcine teschovirus‐1 2A peptide (P2A), followed by GFP to permit identification of transduced cells (AAV8‐CAG‐Adipo‐P2A‐GFP; WZ Biosciences). Control mice received AAV8‐CAG‐GFP. Purified AAV8 particles encoding adiponectin or GFP were diluted in sterile phosphate‐buffered saline (PBS) to a final volume of 100 μL per mouse. Mice were briefly anesthetized with isoflurane (2%–3% induction, 1%–2% maintenance) and placed on a warming pad to promote tail vein dilation. Viral suspensions were administered via the lateral tail vein using a 33‐gauge Hamilton syringe over ~10–15 s to minimize reflux. After injection, gentle pressure was applied to the puncture site for 10–20 s, and mice were monitored until fully ambulatory. The planned viral dose was 1 × 10^11^ vector genomes (vg) per mouse.

### 2.7 Tissue Preparation for Histochemistry

The mice were anesthetized via isoflurane inhalation and blood samples were collected before the mice were perfused with 0.9% saline, followed by 4% paraformaldehyde. The brains were subsequently isolated and postfixed overnight at 4°C. Subsequently, the brains were transferred to a 30% sucrose solution until they sank. Coronal sections with a thickness of 30 μm were prepared with a vibratome (Leica Biosystems, Germany) in a 1‐in‐6 series. These sections were then stored in an antifreeze cryoprotectant solution containing 30% glycerol and 30% ethylene glycol at 4°C until further use.

### 2.8 Immunohistochemistry

Free‐floating brain slices were incubated with antibodies against BrdU, doublecortin (DCX), Ki‐67, and NeuN as previously described (Lee et al. 2021; Yau et al. 2014). To label the survival of adult‐born cells, BrdU was administered via intraperitoneal injection at a dosage of 50 mg/kg for three days prior to the experimental treatments. Brain slices for BrdU labelling were incubated with 2 N HCl at 37°C for 30 min, followed by neutralization with 0.1 M borate buffer (pH 8.5) at room temperature for 15 min. For DCX, Ki‐67, and NeuN staining, antigen retrieval was performed in citric buffer (pH 8.0) at 95°C for 30 min, followed by three washes with 1× phosphate‐buffered saline (PBS). The brain slices were then incubated overnight at room temperature with primary antibodies, including goat anti‐DCX (1:200, sc‐271390, Santa Cruz, USA), rabbit anti‐Ki‐67 (1:1000, ab15580, Abcam, UK) antibodies, and rabbit monoclonal anti‐NeuN (1:1000, ab177487, Abcam, UK). Positive staining was visualized with a VECTASTAIN ABC kit and the DAB peroxidase substrate kits (Vector Laboratories, CA, USA) according to the manufacturer's instructions.

### 2.9 Immunofluorescence

Immunofluorescence staining was conducted according to established protocols (Lee et al. 2021; Yau et al. 2014). After antigen retrieval in citric acid buffer (pH 6.0) and subsequent washes with 1X PBS, the sections were incubated with primary antibodies overnight. The sections were subsequently incubated with anti‐mouse (1:200, TI‐2000, Vector Laboratories, CA, USA) and anti‐rabbit (1:200, TI‐1000, Vector Laboratories, CA, USA) antibodies for 2 h at room temperature. The sections were then cover‐slipped with an anti‐fade mounting medium containing DAPI (Cat No. P0131; Beyotime). Immunofluorescence signals were visualized with a Nikon AXE laser confocal microscope (Nikon, Japan).

### 2.10 Quantification of BrdU, Ki‐67, and DCX‐Positive Cells

Positive cells labeled BrdU, Ki‐67, and DCX were quantified in sections containing the hippocampus from bregma −1.30 to −3.80 mm in a sample blinded manner. The quantification of positive cells was limited to the dentate subgranular zone and granular cell layer while excluding those located in the uppermost focal plane (Pinar et al. 2018). The subgranular zone is defined as the area within approximately two cell bodies (approximately 20 μm) from the inner edge of the molecular layer (Pinar et al. 2018).

### 2.11 Immunofluorescence Co‐Labeling

Co‐labeling PP2A and adiponectin receptor was conducted by using Tyramide Signal Amplification (TSA) Multiplex Immunofluorescence Detection kit (RC0086, Record Bio) according to the manual. The brain sections were incubated with rabbit anti‐PP2Ac antibody (1:200, #2038S; CST) overnight at 4°C. After washing, the sections were incubated with HRP‐conjugated anti‐rabbit secondary antibody (1:200, #7076P2; CST) followed by incubation with tyramide‐570 (TYR‐570, RC0086‐RC001; Record Bio). Images of this channel were quickly captured at low power as a positive control. After blocking antibody‐HRP complexes, the sections were incubated with rabbit anti‐AdipoR1 antibody (1:100, Ab70362; Abcam) or anti‐AdipoR2 antibody (1:200, Ab223752; Abcam) overnight at 4°C. The sections were then incubated with HRP‐conjugated anti‐rabbit secondary antibody (1:200, #7076P2; CST) followed by incubation with tyramide‐520 (RC0086‐RC004; Record Bio). The sections were cover‐slipped with an anti‐fade mounting medium containing DAPI (Cat No. P0131; Beyotime). Immunofluorescence signals were visualized using a Nikon AXE laser confocal microscope (Nikon, Japan).

### 2.12 Western Blotting Analysis

Hippocampal tissues were lysed in RIPA buffer (Beyotime, Shanghai, China) supplemented with protease and phosphatase inhibitors (Thermo Scientific, USA). The total protein concentrations were determined with a BCA protein assay kit (Thermo Fisher Scientific Inc.). Brain tissue proteins (30 μg) were subjected to SDS–PAGE and subsequently transferred to polyvinylidene fluoride (PVDF) membranes. After blocking with 5% bovine serum albumin (BSA) (Macklin, Shanghai, China), the membranes were incubated with the following primary antibodies overnight at 4°C: p‐Tau S404 (1:1000, Abcam, #ab92676); p‐Tau S396 (1:1000, Abcam, #ab109390); Tau‐5 (1:1000, Abcam, #ab80579); PP2Ac (1:1000, CST, #2038S); PME‐1 (1:1000, Santa Cruz, #sc‐25278); LCMT1 (1:1000, Santa Cruz, #sc‐81609); GSK‐3β (1:1000, CST, #12456S); GAPDH (1:5000, Genetex, #GTX100118); and α‐tubulin (1:1000, Invitrogen, #62204). The membranes were subsequently incubated with anti‐mouse (1:2000, CST, #7076P2) and anti‐rabbit (1:2000, CST, #7074S) secondary antibodies for 2 h at room temperature. Immunoreactive bands were visualized and quantified with a ChemiDoc imaging system (Bio‐Rad, California, USA).

### 2.13 PP2A Activity Assay

To evaluate PP2A activity in the hippocampus, taserine/threonine phosphatase assay system kit V2460 (Promega, Madison, WI, USA) was used following the manufacturer's instructions (Lei et al. 2022). Endogenous free phosphate was extracted from brain tissues using spin columns and phosphatase‐free storage buffer (1 g of tissue with 3 mL of buffer). Triplicate protein samples (5 μg each) were incubated with a chemically synthesized phosphopeptide (RRA (pT)VA), an optimal substrate for PP2A, PP2B, and PP2C, in a buffer optimized for PP2A activity. Cation‐dependent PP2B and PP2C were inhibited during the 30‐min incubation at 32°C (Lei et al. 2022). The released phosphate was quantified by measuring the absorbance of the molybdate‐malachite green–phosphate complex at 630 nm. PP2A activity was calculated as the release of phosphate per μg of protein per minute (pmol/μg/min).

### 2.14 Quantitative Reverse Transcription PCR

Total RNA was extracted using a Total RNA Kit (R6934‐02, Omega, USA). RNA was reverse transcribed to cDNA using the Hifair AdvanceFast 1st Strand cDNA Synthesis Kit (11149ES60, Yeasen, Shanghai, China). PCR amplification of target genes was performed using a PCR thermal cycler (Thermo Fisher Scientific, USA) and SYBR Green (11203ES08, Yeasen, Shanghai, China) according to the manufacturer's protocol. The sequences of primers used are provided in Table S2.

### 2.15 Adiponectin Enzyme‐Linked Immunosorbent Assay (ELISA)

Serum adiponectin levels were quantified using a mouse adiponectin ELISA kit (Immunodiagnostics, University of Hong Kong, Hong Kong). Adiponectin levels in the hippocampus were measured using mouse adiponectin ELISA kits (AdipoGen Life Sciences, Switzerland) as previously described (Yau et al. 2014).

### 2.16 Statistical Analysis

The Shapiro–Wilk test was used to test the normality of the data. Parametric data were analyzed via Student's *t*‐test for comparing two groups and one‐way ANOVA with Tukey's post hoc test for comparing multiple groups using GraphPad Prism 9.4.0 software (GraphPad Software, San Diego, CA, USA). Non‐parametric data were analyzed via the Kruskal–Wallis test with Dunn's multiple comparisons for multiple‐group comparisons or the Kolmogorov–Smirnov test for two‐group comparisons. The data are presented as the means ± SEMs. Statistical significance was determined at a significance level of *p* < 0.05.

## 3 Results

### 3.1 Physical Exercise Restores Behavioral Deficits Induced by Chronic Stress

**FIGURE 1:**
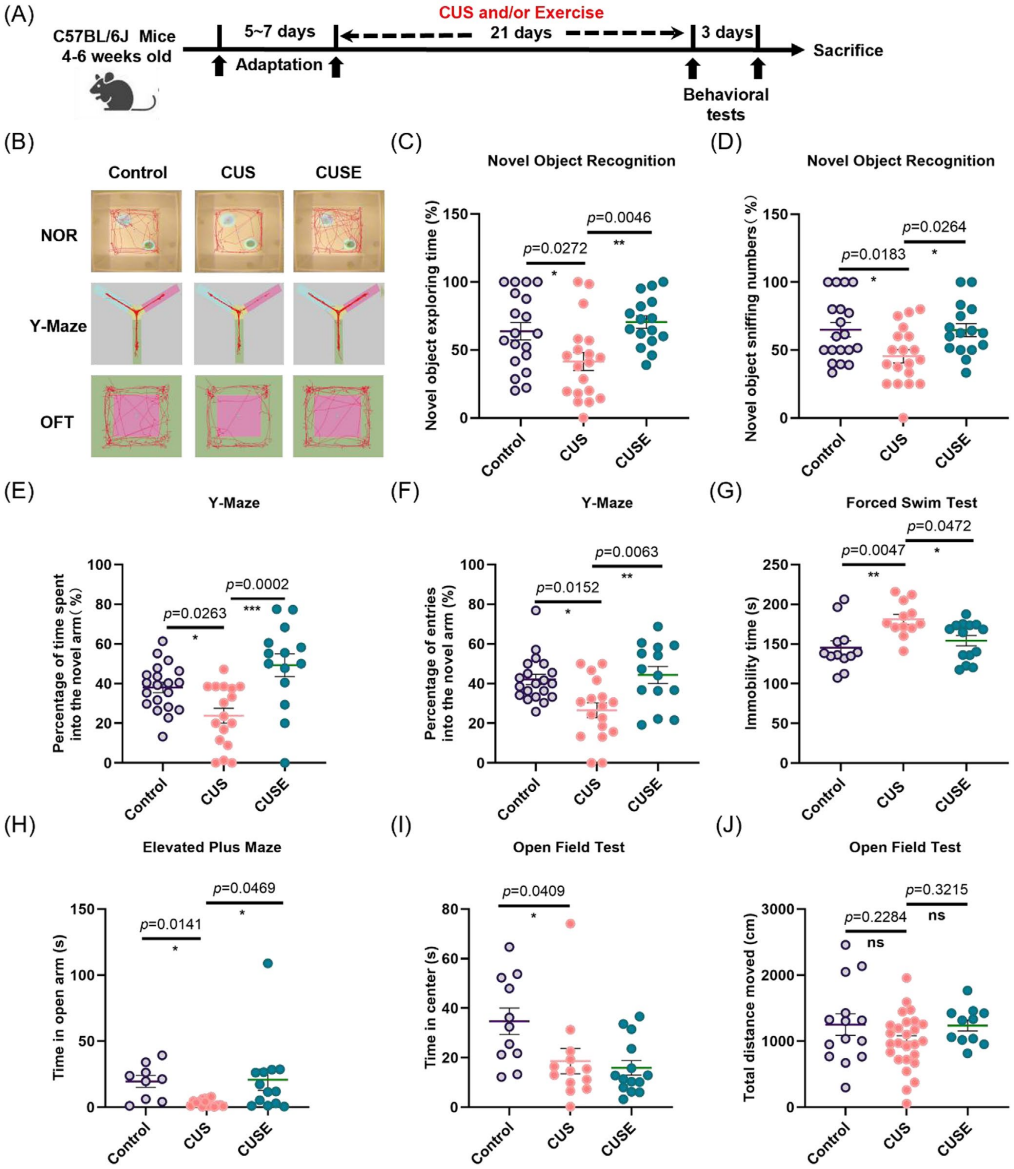
Physical exercise restored cognitive behavioral deficits in stressed mice. (A) Experiment timeline with 3‐week voluntary running and chronic unpredictable stress (CUS) paradigm, followed by a battery of behavioral tests. (B) Representative traces of animal behavioral performance in the novel object recognition (NOR) test, Y maze, and open field test (OFT). (C) Percentage of time spent and (D) numbers of sniffing to the novel object in the NOR test. *p < 0.05, **p < 0.01; *N* = 19, 19, and 16 mice in Control, CUS, and CUSE groups, respectively. (E) Percentage of time spent and (F) number of visits to the novel arm in the Y‐maze. *p < 0.05, **p < 0.01, and ***p < 0.001; *N* = 20, 17 and 14 mice in Control, CUS, and CUSE groups, respectively. (G) Immobility time in the forced swim test. *p < 0.05, **p < 0.01; *N* = 12, 12 and 14 mice in Control, CUS, and CUSE groups, respectively. (H) Time spent in the open arms of the elevated plus maze (EPM) test. *p < 0.05; *N* = 9, 16, and 13 mice in Control, CUS, and CUSE groups, respectively. (I) Total time spent in the central area and (J) the total traveling distance in the open field test. *p < 0.05; *N* = 11, 13, and 14 mice in Control, CUS, and CUSE groups, respectively. “ns” means non‐significant difference. Data were analyzed using one‐way analysis of variance followed by Tukey's post hoc test for intergroup comparison and the Kruskal–Wallis test with Dunn's multiple comparisons for multiple‐group comparisons. All data are presented as mean ± standard error of means (SEM).

To demonstrate the positive effects of physical exercise on the behavioral deficits induced by chronic stress, mice were subjected to a battery of behavioral assessments after a 3‐week voluntary running (Figure 1A). Voluntary running significantly improved working memory in stressed mice in the NOR test, as evidenced by an increase in exploration time spent on the novel object (Figure 1B,C: *F*_2_,_51_ = 6.275, *p* = 0.0037) and the number of times the mice sniffed the novel object (Figure 1B,D: *F*_2_,_51_ = 5.129, *p* = 0.0093).

In the Y‐maze task, stressed mice without running exhibited a significant reduction in time spent in the novel arm (Figure 1E, *p* < 0.05 vs. the control) and the number of visits to the novel arm (Figure 1F, *p* < 0.05 vs. the control) when compared with the non‐stressed and non‐exercised control, indicating impaired hippocampus‐dependent spatial recognition learning and memory functions in stressed mice (Figure 1B,E,F). Conversely, exercised mice spent more time in the novel arm (Figure 1B,E: *F*_2_,_48_ = 9.837, *p =* 0.0003) and made more visits to the novel arm (Figure 1B,F: Kruskal–Wallis statistic = 11.63, *p* = 0.0030), indicating the effectiveness of physical exercise in reversing chronic stress‐impaired hippocampal spatial memory.

In stressed mice, voluntary running effectively reduced depression‐like behavior (Figure 1G, immobility time: Kruskal–Wallis statistic = 10.10, *p* = 0.0064) in the FST and anxiety‐like behavior in the EPM (Figure 1H, time spent in the open arm: Kruskal–Wallis statistic = 9.949, *p* = 0.0069), but showed no significant effect in the OFT (Figure 1I, time spent in the center: Kruskal–Wallis statistic = 9.166, *p* > 0.9999, CUS vs. CUSE). Moreover, the results revealed no significant differences in locomotor activity among the groups (Figure 1B,J: *F*_2_,_48_ = 1.859, *p* = 0.1669; Figure S1B: *F*_2_,_46_ = 2.218, *p* = 0.1204). These findings collectively suggest that voluntary running rescues cognitive impairments and mitigates the increase in depression−/anxiety‐like behaviors in stressed mice.

### 3.2 Physical Exercise Reduces Hippocampal p‐Tau Levels in Association With Increased Adiponectin Levels

**FIGURE 2:**
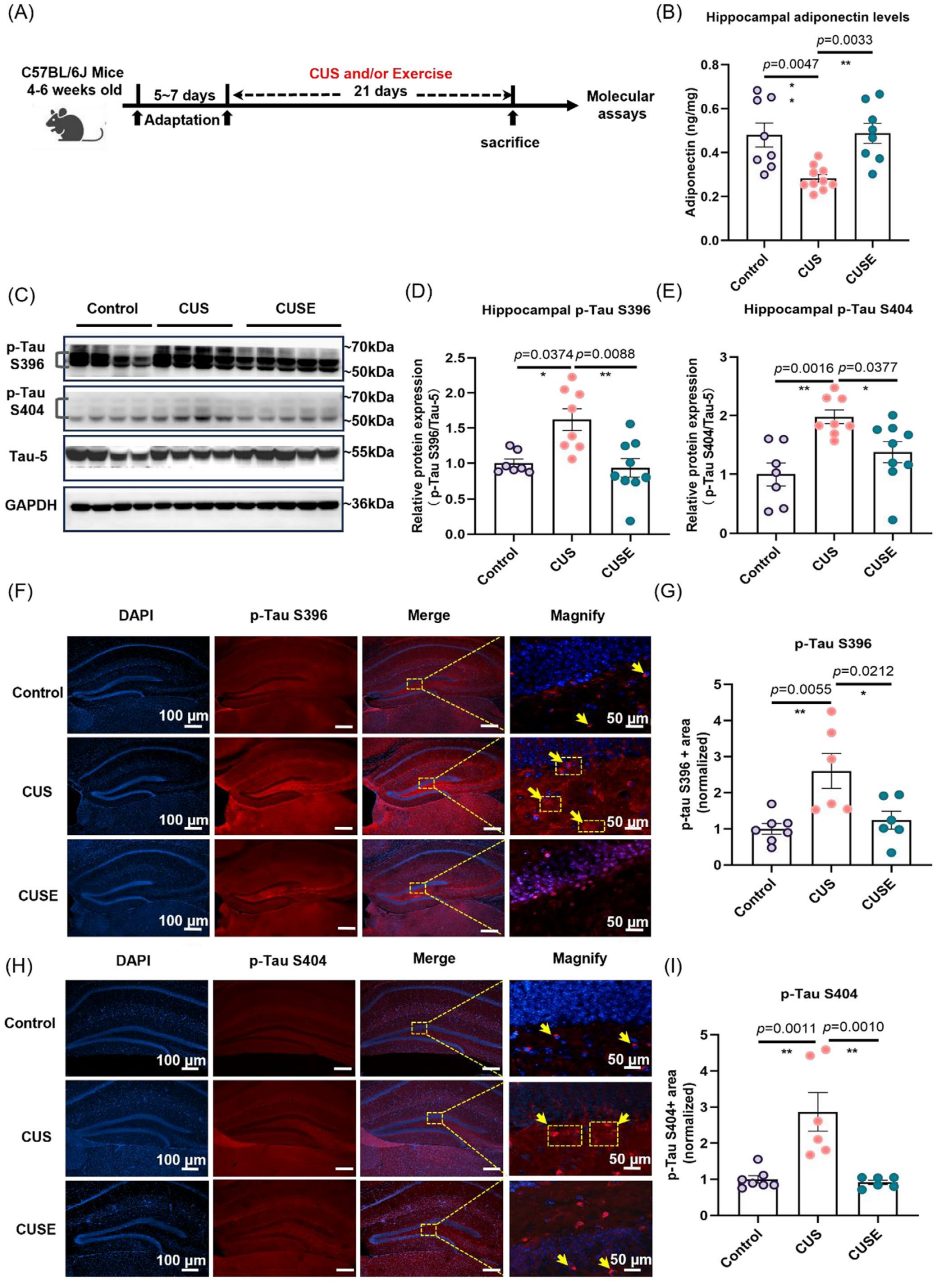
Physical exercise restored hippocampal adiponectin levels and reduced Tau hyperphosphorylation in stressed mice. (A) Experimental timeline showing 3‐week voluntary running and chronic unpredictable stress paradigm, followed by the tissue collection. (B) Stress decreased hippocampal adiponectin levels, which were restored by voluntary running. *p < 0.05, **p < 0.01; *N* = 8, 10, and 8 mice in Control, CUS, and CUSE groups, respectively. (C) Representative western blotting images. Voluntary running counteracted chronic stress‐induced hippocampal tau phosphorylation at (D) p‐Tau S396 and (E) p‐Tau S404. *p < 0.05; **p < 0.01; *N* = 7, 8, and 9 mice in Control, CUS, and CUSE groups, respectively. (F and H) Representative images of immunofluorescence staining for p‐Tau S396 (red) and p‐Tau S404 (red). Quantification of immunofluorescence intensity of (G) p‐Tau S396 and (I) p‐Tau S404 (red) in hippocampal dentate gyrus regions. Scale bar, 100 μm and 50 μm. *p < 0.05, **p < 0.01. *N* = 48–54 slices from 6 mice per genotype. Data were analyzed using one‐way analysis of variance followed by Tukey's post hoc test for intergroup comparison and the Kruskal–Wallis test with Dunn's multiple comparisons for multiple‐group comparisons. All data are presented as mean ± SEM.

Previous studies have demonstrated the critical role of adiponectin in mediating the beneficial effects of physical exercise on mood and cognitive functions (Lee et al. 2021; Yau et al. 2014). We further tested whether adiponectin is required for physical exercise to reduce hippocampal Tau phosphorylation (Figure 2A). Increase in Tau phosphorylation at Ser396–404 sites have been recognized as the earliest event in AD (Mondragón‐Rodríguez et al. 2014). Previous study has shown that S396/S404 phosphorylation in the hippocampus is linked to AD pathogenesis and cognitive decline (Lei et al. 2023). We therefore focused on the serine 396 (S396) and serine 404 (S404), which are well‐established markers of early AD pathology (Mondragón‐Rodríguez et al. 2014) and are associated with the formation paired helical filaments and neurofibrillary tangles (Cantrelle et al. 2021; Drummond et al. 2020; Torres et al. 2021) in the hippocampus. Moreover, PP2A induces the phosphorylation of Tau‐S396, and S404 (Sontag et al. 1996). We therefore tested on PP2A's action in reducing these two Tau phosphorylation sites.

Voluntary running mitigated the decrease in hippocampal adiponectin levels in stressed mice (Figure 2B: *F*_2_,_23_ = 9.078, *p* = 0.0012) and attenuated stress‐induced Tau hyperphosphorylation (S396 and S404) in stressed mice (Figure 2C: Kruskal–Wallis statistic = 10.23, *p* = 0.0060; Figure 2D: *F*_2_,_21_ = 8.464, *p* = 0.0020; and Figure 2E). Furthermore, immunofluorescence staining confirmed the beneficial effects of voluntary running on reducing hippocampal p‐Tau levels in stressed mice (Figure 2F,H: *F*_2_,_16_ = 7.595, *p* = 0.0048; Figure 2G,I: *F*_2_,_16_ = 13.12, *p* = 0.0004). These data indicate that voluntary running effectively counteracts hippocampal Tau hyperphosphorylation in stressed mice.

### 3.3 Adiponectin Deficiency Induces Behavioral Deficits in an Association With Tau Hyperphosphorylation

**FIGURE 3:**
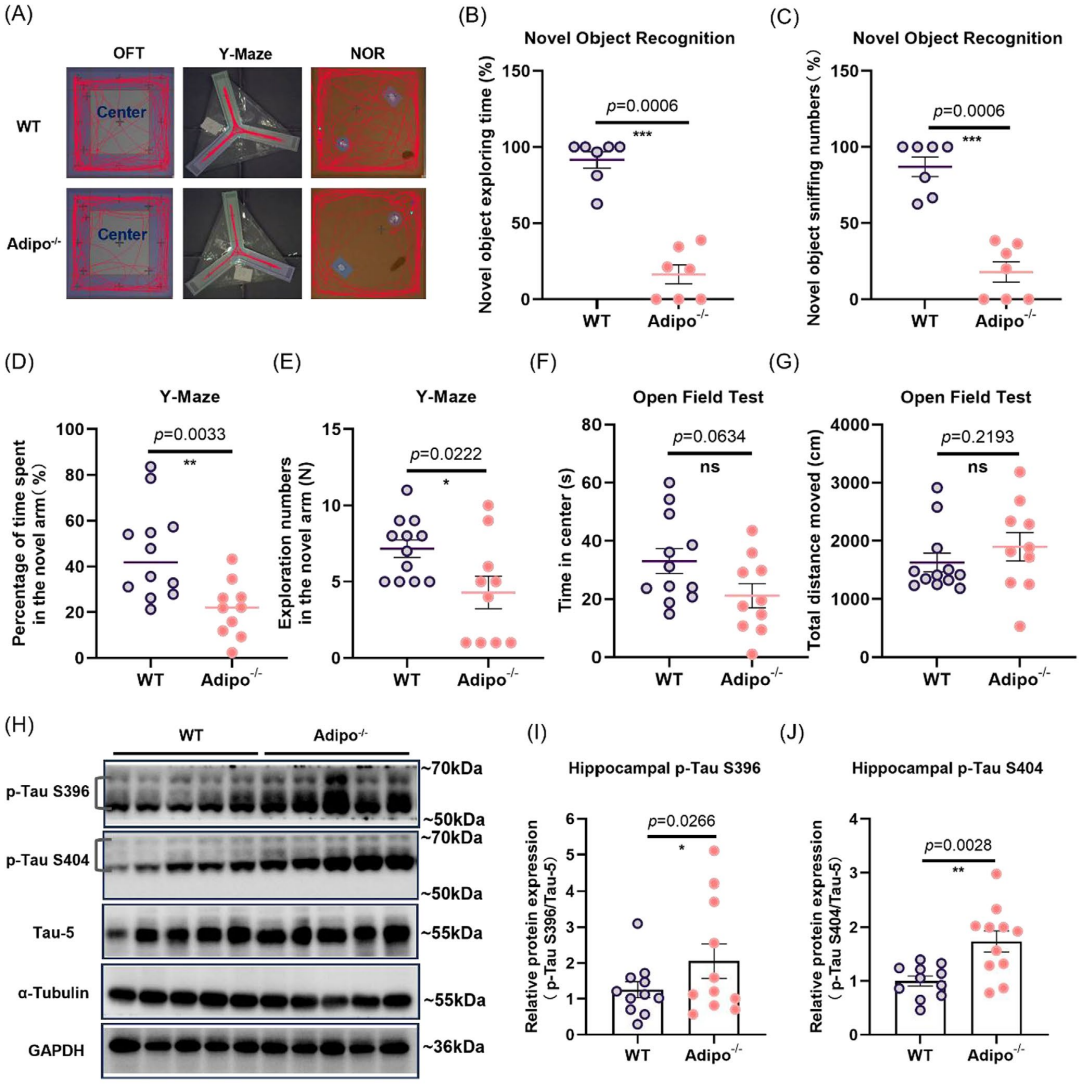
Adiponectin deficiency impaired memory performance in association with increased Tau phosphorylation in the hippocampus. (A) Representative behavioral traces of behavioral tests in adiponectin knockout (*Adipo* ^−/−^) and wild type mice. (B) The percentage of time spent sniffing the novel object and (C) percentage numbers of sniffing the novel object in the novel object recognition task in adiponectin knockout mice. ***p < 0.001; *N* = 7 mice per genotype. (D) The percentage of time spent and (E) number of visits to the novel arm in the Y‐maze. *p < 0.05, **p < 0.01; *N* = 12 and 10 mice in WT and *Adipo* ^−/−^ groups, respectively. (F) Time spent in the center and (G) total traveling distance in the open field test. *N* = 12 and 10 mice in WT and *Adipo* ^−/−^ groups, respectively. (H) Representative western blotting images. (I) Quantification of p‐Tau S396 and (J) p‐Tau S404 protein expression in the hippocampus. *p < 0.05; *N* = 11 mice per genotype. “ns” means non‐significant difference. Data were analyzed by the Student's *t*‐test or Kolmogorov–Smirnov test for two‐group comparisons. All data are presented as mean ± SEM.

To further investigate the critical role of adiponectin in reducing Tau phosphorylation in exercised mice, *Adipo* ^−/−^ mice were used. *Adipo* ^−/−^ mice exhibited a significant impairment in working memory performance, as indicated by a significantly lower discrimination index (Figure 3B: Kolmogorov–Smirnov *D* = 1.000, *p* = 0.0006) and fewer sniffs when exploring the novel object in the NOR test (Figure 3C: Kolmogorov–Smirnov *D* = 1.000, *p* = 0.0006). Additionally, adiponectin knockout significantly reduced the exploratory time spent in the novel arm in the Y‐maze test (Figure 3D: *t*_20_ = 3.335, *p* = 0.0033; Figure 3E: *t*_20_ = 2.480, *p* = 0.0222). Adiponectin deficiency marginally increased anxiety‐like behavior (Figure 3F: *t*_20_ = 1.966, *p* = 0.0634), whereas the mice displayed locomotor activity comparable to that of control mice (Figure 3G: Kolmogorov–Smirnov *D* = 0.4500, *p* = 0.2193). Notably, adiponectin deficiency increased the p‐Tau protein levels at both S396 (Figure 3I: *t*_8_ = 4569, *p* = 0.0018) and S404 sites (Figure 3J: *t*_8_ = 2.795, *p* = 0.0234). Collectively, these findings showed that adiponectin deficiency increases Tau phosphorylation in the hippocampus, suggesting a link between Tau hyperphosphorylation and decreased adiponectin levels.

Next, we examine whether adiponectin supplementation rescues hippocampal deficits in *Adipo* ^−/−^ mice (Figure S3A). In adiponectin knockout mice, adiponectin overexpression increased serum adiponectin levels (Figure S3B: *t*_4_ = 2.785, *p* = 0.0496) and rescued memory deficit in NOR (Figure S3D exploration time: *F*_2,20_ = 11.71, *p* = 0.0004; Figure S3E novel object sniffing time: *F*_2,20_ = 5.469, *p* = 0.0127), and spatial memory impairment in Y maze test (Figure S3F time spent in novel arm: *F*_2,20_ = 12.83, *p* = 0.0003; Figure S3G the number of entries into the novel arm: *F*_*2,20*_ = 8.596, *p* = 0.0020). Furthermore, adiponectin overexpression reduced anxiety‐like behaviors in the OFT (Figure S3H: *F*_2,20_ = 5.569, *p* = 0.0120) but showed no effect in the total distance traveled (Figure S3I: *F*_2,20_ = 0.05621, *p* = 0.9455). In addition, adiponectin overexpression attenuated the reduction in PP2A activity (Figure S3C: *F*_2,20_ = 26.67, *p* < 0.0001) and reduced Tau protein phosphorylation at S396 (Figure S3J: *F*_2,20_ = 5.076, *p* = 0.0165) and S404 (Figure S3J: *F*_2,20_ = 5.567, *p* = 0.0120) in Adipo‐/‐ mice. These data confirmed the critical role of adiponectin in rescuing cognitive deficits and regulating hippocampal PP2A activity and Tau protein phosphorylation.

### 3.4 Adiponectin Is Required for Physical Exercise to Reduce Hippocampal Tau Hyperphosphorylation

**FIGURE 4:**
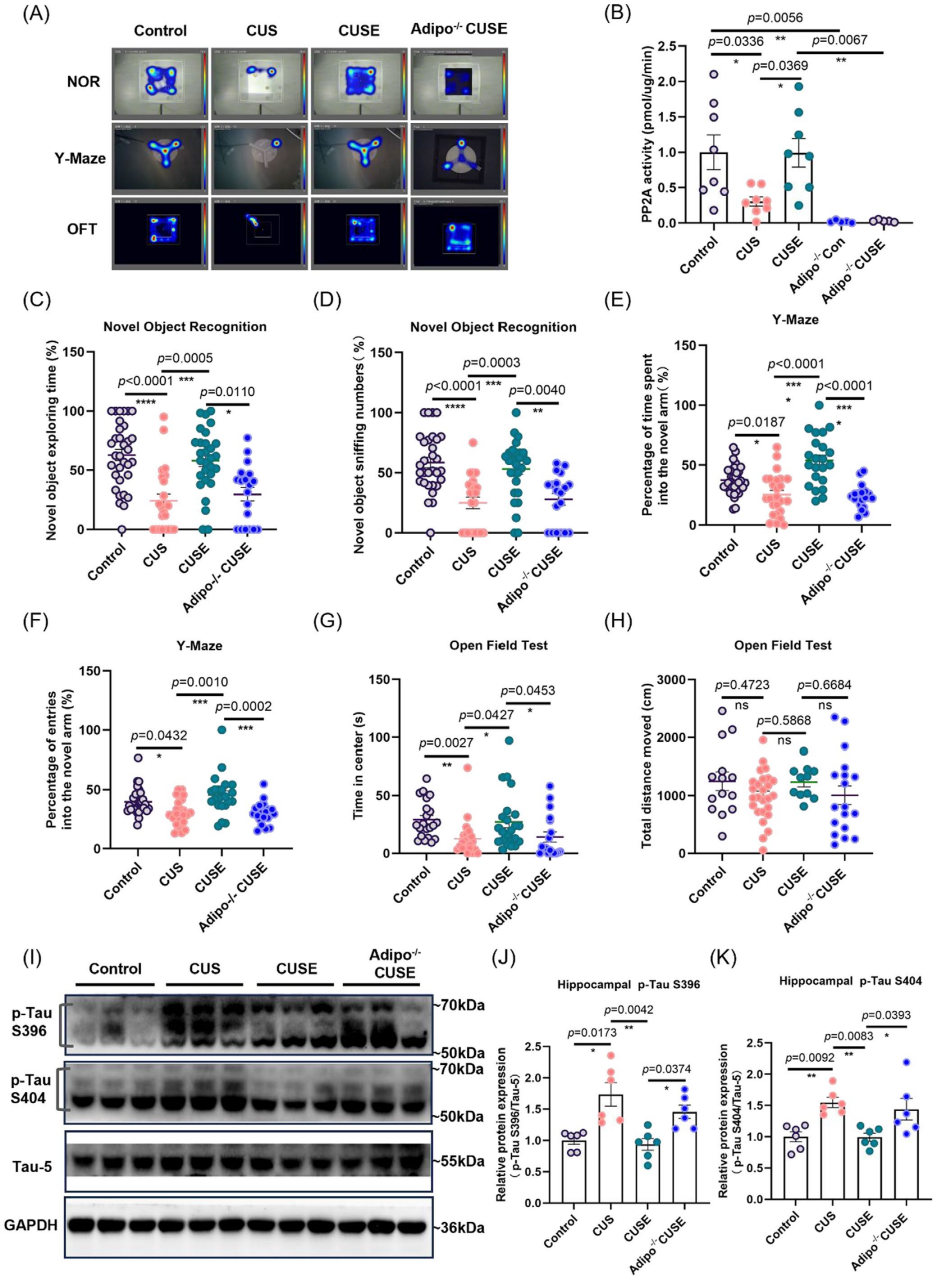
Adiponectin deficiency abolished the effects of voluntary running in restoring behavioral deficits, reducing hippocampal p‐Tau protein levels, and increasing PP2A activity in stressed mice. (A) Representative behavioral traces in behavioral tests. (B) PP2A activity assay. (C) The percentage of time spent in sniffing the novel object and (D) percentage numbers of sniffing the novel object in novel object recognition task. *p < 0.05, **p < 0.01, ***p < 0.001, and ****p < 0.0001; *N* = 33, 23, 29, and 19 mice in Control, CUS, CUSE, and *Adipo* ^−/−^ CUSE groups, respectively. (E) Percentage of time spent and (F) number of visits to the novel arm in the Y‐maze. *p < 0.05, ***p < 0.001 and ****p < 0.0001; *N* = 34, 25, 22, 20 in Control, CUS, CUSE, and *Adipo* ^−/−^ CUSE groups, respectively. (G) Time spent in the center and (H) total traveling distance in the open field test. *p < 0.05, **p < 0.01; *N* = 21, 22, 23, and 18 mice in Control, CUS, CUSE and *Adipo* ^−/−^ CUSE groups, respectively. (I) Representative protein expression level of (J) p‐Tau S396 and (K) p‐Tau S404 in hippocampus. *p < 0.05, **p < 0.01; *N* = 6 mice per genotype group. “ns” means non‐significant difference. Data were analyzed using One‐way analysis of variance followed by Tukey's post hoc test for intergroup comparison and the Kruskal–Wallis test with Dunn's multiple comparisons for multiple‐group comparisons. All data are presented as mean ± SEM.

Next, we examined whether adiponectin mediates the effects of physical exercise on reducing hippocampal p‐Tau levels and alleviating cognitive deficits in stressed mice. Our result showed that adiponectin deficiency abolished the rescuing effects of voluntary running on memory impairment, as *Adipo* ^−/−^ mice with running showed significant decreases in time spent exploring the novel object and number of times sniffing the novel object in NOR (Figure 4C: Kruskal–Wallis statistic = 31.01, *p* < 0.0001; Figure 4E: Kruskal–Wallis statistic = 31.32, *p* < 0.0001 and Figure 4A), and absence of increase in exploration time and the number of entries into the novel arm in the Y‐maze task (Figure 4E: *F*_3_,_97_ = 18.29, *p* < 0.0001; Figure 4F: Kruskal–Wallis statistic = 24.67, *p* < 0.0001 and Figure 4A). In addition, adiponectin deficiency abolished the anxiolytic effects of voluntary running in the stressed mice, as indicated by a reduced duration in the center zone (Figure 4G: Kruskal–Wallis statistic = 19.59, *p* = 0.0002 and Figure 4A). However, there was no significant effect on locomotor activity in the OFT (Figure 4H: *p* = 0.4723, Control vs. CUS; *p* = 0.5868, CUS vs. CUSE; *p* = 0.6684, CUSE vs. *Adipo* ^−/−^ CUSE and Figure 4A). In summary, these data indicate that adiponectin is indispensable for running‐improved cognitive performance in stressed mice.

We then examined whether adiponectin is required for voluntary running to reduce stress‐induced Tau hyperphosphorylation in the hippocampus. As expected, adiponectin deficiency abolished the effect of voluntary exercise on counteracting stress‐induced Tau hyperphosphorylation (Figure 4J for S396: Kruskal–Wallis statistic = 16.93, *p* = 0.0007; Figure 4K for S404: *F*_3_,_21_ = 7.229, *p* = 0.0016; Figure 4I), suggesting the critical role of adiponectin in mediating the beneficial effects of running. Most importantly, adiponectin deficiency significantly decreased PP2A activity in the hippocampus (Figure 4B: *F*_4_,_29_ = 7.878, *p* = 0.0002), indicating its function in regulating hippocampal PP2A activity.

### 3.5 Adiponectin Is Required for Physical Exercise to Increase Adult Hippocampal Neurogenesis in Stressed Mice

**FIGURE 5:**
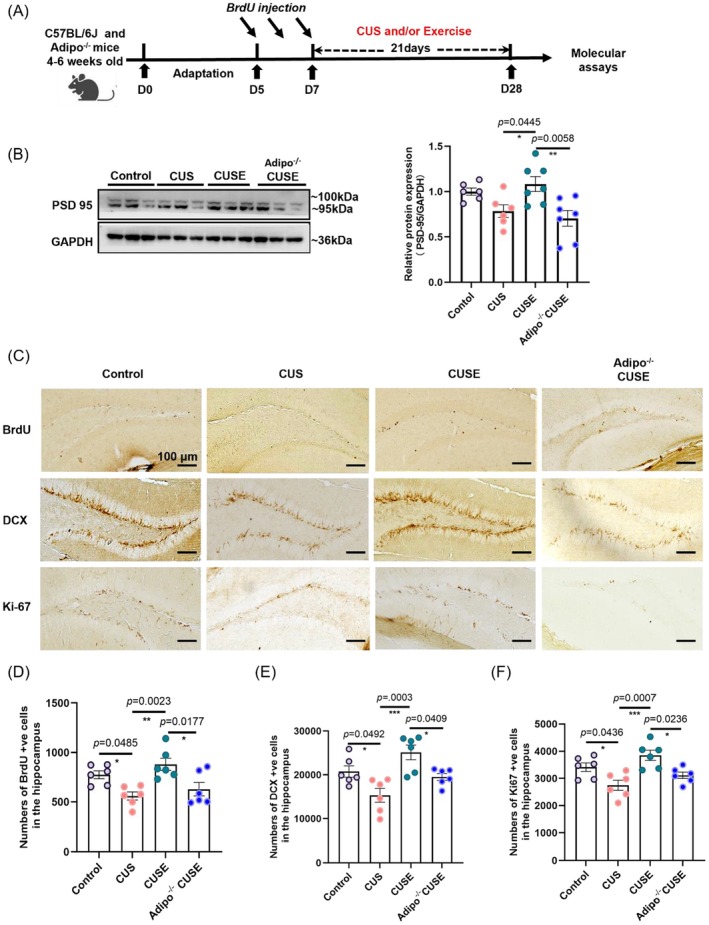
Adiponectin deficiency abolished voluntary running to restore adult hippocampal neurogenesis in stressed mice. (A) Experiment timeline with 3‐week voluntary running and CUS paradigm in *Adipo* ^−/−^ mice, followed by the tissue collection. (B) Representative expression level of post‐synaptic protein PSD‐95 in WT and *Adipo* ^−/−^ mice. (C) Representative images of BrdU, Ki‐67, and DCX immunostaining. (D) Quantification of BrdU^+^ surviving adult‐born cells and (E) doublecortin (DCX)^+^ immature neurons in hippocampal dentate gyrus and (F) Ki‐67^+^ proliferating cells. *p < 0.05, **p < 0.01, ***p < 0.001; *N* = 48–54 slices from 6 mice per genotype. Scale bar, 100 μm. *p < 0.05, **p < 0.01; *N* = 6 and/or 7 mice per genotype. Data were analyzed using one‐way analysis of variance followed by Tukey's post hoc test for intergroup comparison and the Kruskal–Wallis test with Dunn's multiple comparisons for multiple‐group comparisons. All data are presented as mean ± SEM.

To examine changes of hippocampal structural plasticity, we examined the number of surviving newborn cells (BrdU^+^), proliferating cells (Ki‐67^+^), and immature neurons (DCX^+^) (Figure 5C–F). Our findings revealed that chronic stress significantly decreased the number of BrdU‐, Ki‐67‐, and DCX‐positive cells (Figure 5D: BrdU, *F*_3_,_20_ = 7.110, *p* = 0.0019; Figure 5E: DCX, *F*_3_,_20_ = 8.680, *p* = 0.0007; Figure 5F: Ki‐67, *F*_3_,_20_ = 8.027, *p* = 0.0010), which could be restored by voluntary running. However, the beneficial effects of running were abolished by adiponectin deficiency (Figure 5C–F). Furthermore, Western blotting revealed that voluntary running restored hippocampal levels of PSD‐95 in stressed mice (Figure 5B: *F*_3_,_22_ = 5.954, *p* = 0.0445, CUS vs. CUSE in WT mice), which was abolished by adiponectin deficiency (Figure 5B: *F*_3_,_22_ = 5.954, *p* = 0.0058, CUSE vs. CUSE in *Adipo* ^−/−^ mice). Collectively, these results suggest that voluntary running requires adiponectin to restore hippocampal plasticity in stressed mice.

### 3.6 Adiponectin Increases PP2A Activity to Reduce Tau Hyperphosphorylation

**FIGURE 6:**
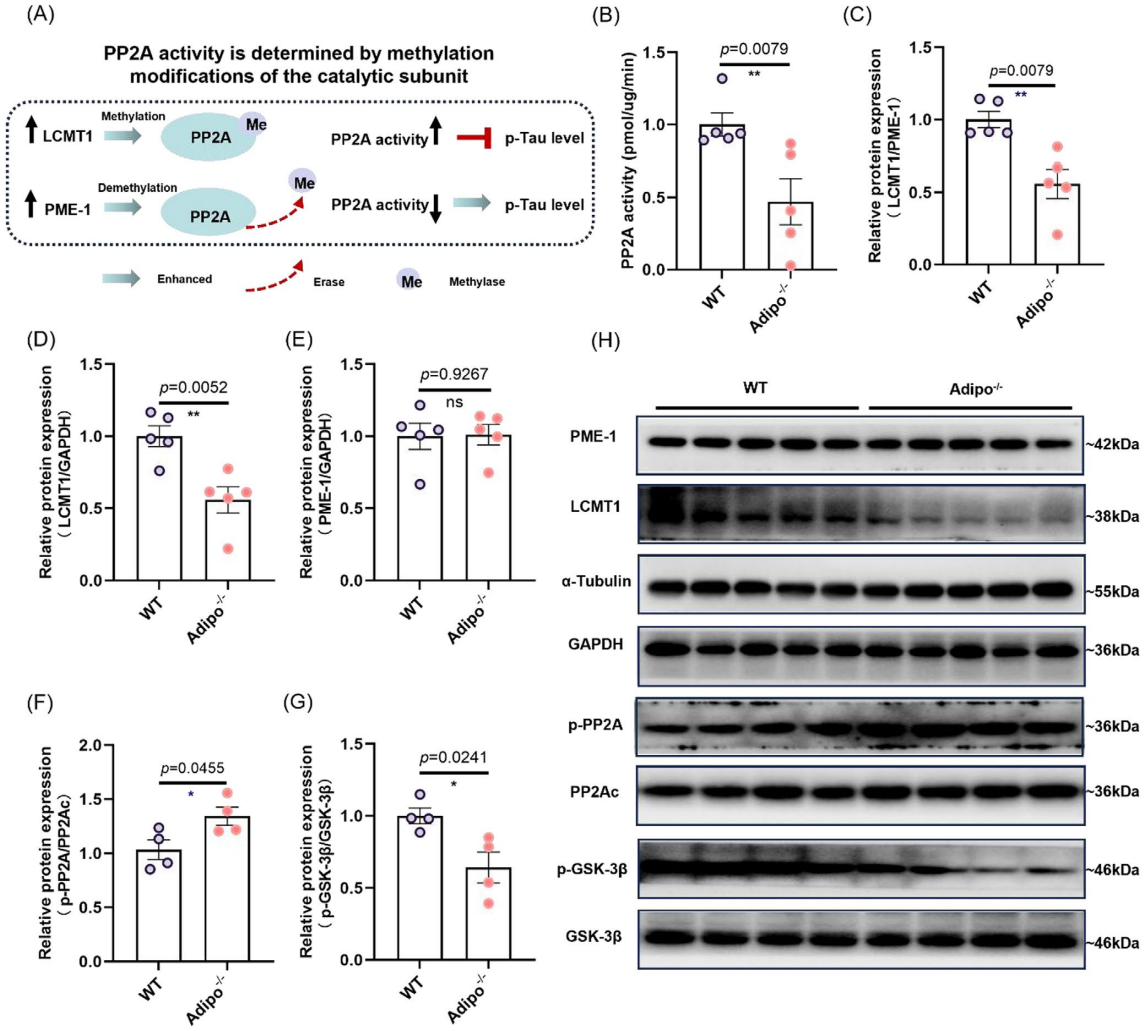
Adiponectin knockout decreased PP2A activity in the hippocampus. (A) Schematic showing that PP2A activity is determined by methyltransferase LCMT1 and demethyltransferase PME‐1, which control p‐Tau levels. (B) Decreased PP2A activity *Adipo* ^−/−^ mice compared to wild type mice, **p < 0.01. (C) Quantifications of the LCMT1/PME‐1 expression ratio, **p < 0.01. (D) Expression level of LCMT1. **p < 0.01. (E) Expression level of PME‐1 (F) p‐PP2A (Tyr307), and (G) p‐GSK‐3β (Ser9) in the hippocampus. *p < 0.05. (H) Representative western blot images. *N* = 4 or 5 mice per genotype. “ns” means non‐significant difference. Data were analyzed by the Student's *t*‐test or Kolmogorov–Smirnov test for two‐group comparisons. All data are presented as mean ± SEM.

Hippocampal PP2A plays an important role in the reduction of Tau phosphorylation by physical exercise in stressed mice (W. Zhang et al. 2021). PP2A activity can be regulated by PP2A methylation (Figure 6A) (Lei et al. 2022; Rasool et al. 2023; Ye et al. 2024). Leucine carboxyl methyltransferase 1 (LCMT1) promotes PP2A methylation and enhances holoenzyme assembly and activity, whereas protein phosphatase methylesterase 1 (PME‐1) demethylates PP2A to decrease PP2A activity (Figure 6A) (Lei et al. 2022; Stanevich et al. 2011; Xing et al. 2008).

To determine whether adiponectin regulates PP2A activity, we examined the protein expression levels of LCMT1, PME‐1, phosphorylated PP2Aα (Tyr307) (p‐PP2A), and phosphorylated GSK‐3β (Ser9) (p‐GSK‐3β), as well as PP2A activity in the hippocampus of *Adipo* ^−/−^ mice. Co‐labelling confirmed that PP2Ac positive cells express both AdipoR1 and AdipoR2 in the hippocampus (Figure S4A,B). Adiponectin deficiency significantly decreased hippocampal PP2A activity (Figure 6B: Kolmogorov–Smirnov *D* = 1.000, *p* = 0.0079). In addition, the ratio of LCMT1 to PME‐1 (Figure 6C: Kolmogorov–Smirnov *D* = 1.000, *p* = 0.0079), two main modifying enzymes of PP2A methylation, was decreased in the *Adipo* ^−/−^ mice, indicating that adiponectin deficiency could decrease this ratio and subsequently decrease PP2A activity to induce Tau hyperphosphorylation. Specifically, adiponectin deficiency decreased the expression of the PP2A methyltransferase LCMT1 (Figure 6D: *t*_8_ = 3.799, *p* = 0.0052 and Figure 6H), though there was no difference in the PME‐1 expression level between WT and *Adipo* ^−/−^ mice (Figure 6E: *t*_8_ = 0.095, *p* = 0.9267 and Figure 6H). Consistent with these findings, adiponectin deficiency increased PP2A phosphorylation (Figure 6F: *t*_6_ = 2.516, *p* = 0.0455 and Figure 6H), but suppressed GSK‐3β phosphorylation (Figure 6G: *t*_6_ = 2.996, *p* = 0.0241 and Figure 6H), suggesting that adiponectin deficiency inhibited PP2A activity and increased GSK‐3β activity. In summary, these results suggest that adiponectin regulates Tau phosphorylation possibly through regulating PP2A activity.

### 3.7 PP2A Activity Is Essential for Exercise to Reduce p‐Tau Levels and Improve Behavioral Deficits

**FIGURE 7:**
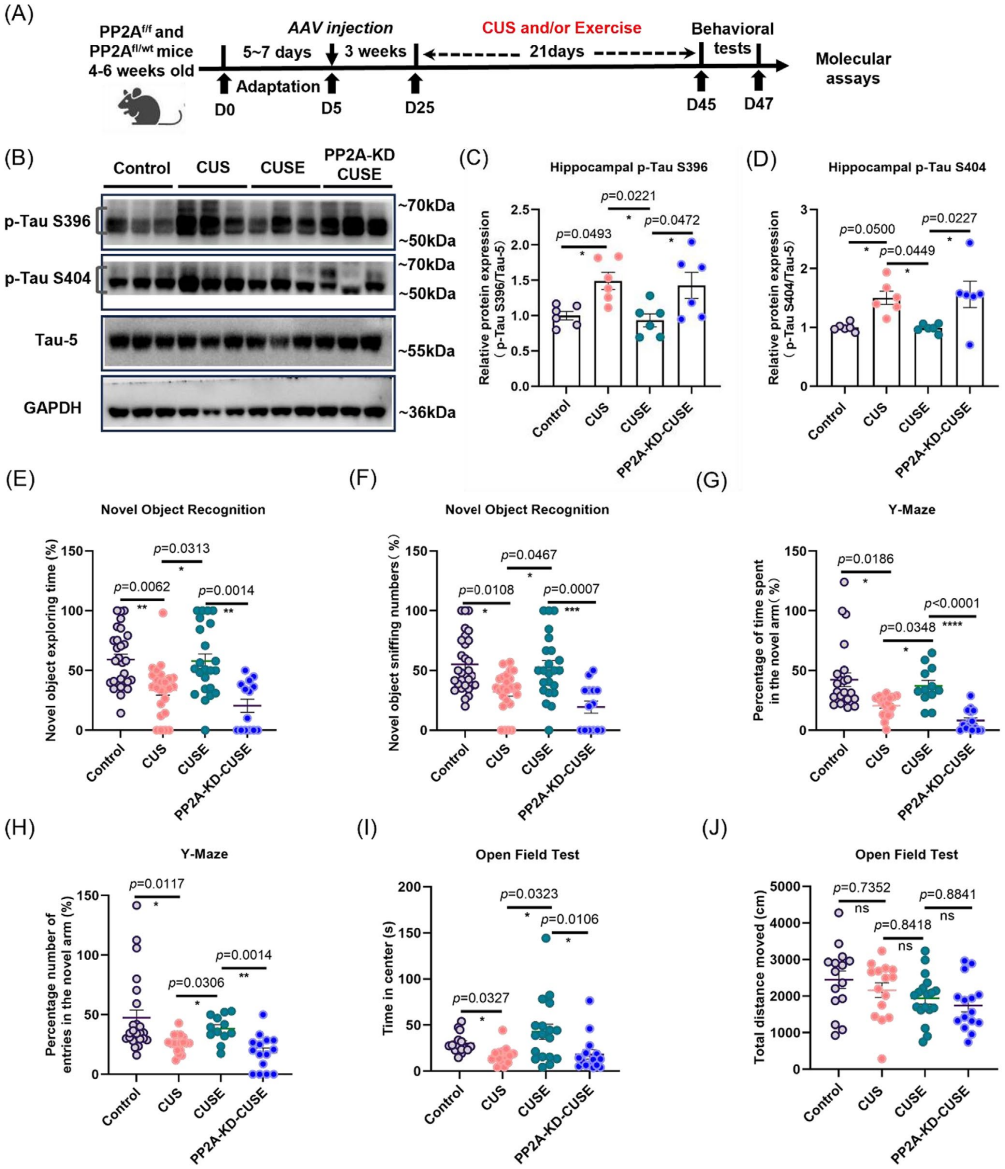
Hippocampal‐specific PP2A knockdown abolished the rescuing effects of voluntary running in stressed mice. (A) Experimental timeline showing AAV injection, followed by 3‐week voluntary running, CUS paradigm, and tissue collection. (B) Representative western blotting images. (C) Western blotting analysis of p‐Tau S396 and (D) p‐Tau S404 proteins in the hippocampus. *p < 0.05; *N* = 6 mice per genotype group. (E) Percentage of time spent in sniffing the novel object and (F) percentage numbers of sniffing the novel object in novel object recognition task. *p < 0.05, **p < 0.01, ***p < 0.001, and ****p < 0.001; *N* = 29, 25, 23, and 14 mice in Control, CUS, CUSE, and PP2A‐KD‐CUSE groups, respectively. (G) Percentage of time spent and (H) number of visits to the novel arm in the Y‐maze. *p < 0.05, **p < 0.01, and ****p < 0.0001; *N* = 24, 18, 12, and 15 mice in Control, CUS, CUSE, and PP2A‐KD‐CUSE groups, respectively. (I) Time spent in the center and (J) total traveling distance in the open field test. *p < 0.05; *N* = 15 or 18 mice in each group. “ns” means non‐significant difference. Data were analyzed using one‐way analysis of variance followed by Tukey's post hoc test for intergroup comparison and the Kruskal–Wallis test with Dunn's multiple comparisons for multiple‐group comparisons. All data are presented as mean ± SEM.

To confirm the role of hippocampal PP2A activity in regulating Tau phosphorylation, hippocampal‐specific PP2A was knocked down (PP2A‐KD) by injecting AAVs carrying Cre‐dependent recombinase into the hippocampal CA1 region of PP2A^f/f^ mice (Figure 7A). Decrease in PP2A expression was validated by qPCR (Figure S2A,B, *t* = 3.740, *p* = 0.0201, PP2A‐KD vs. Control) and Western blotting (Figure S2C,D, *t* = 7.439, *p* = 0.0003, PP2A‐KD vs. Control). Hippocampal PP2A‐KD significantly abolished the effects of voluntary running on reducing p‐Tau protein levels at S396 (Figure 7B,C: *F*_3_,_20_ = 5.382, *p* = 0.0472, CUSE vs. PP2A‐KD‐CUSE), but not S404 (Figure 7B,D: *F*_3_,_20_ = 5.382, *p* = 0.0227, CUSE vs. PP2A‐KD‐CUSE) in stressed mice.

In the behavioral tests, hippocampal PP2A‐KD abolished the effects of voluntary running on restoring working memory deficit induced by chronic stress (Figure 7E: Kruskal–Wallis statistic = 24.78, *p* < 0.05 CUS vs. CUSE; *p* < 0.005 CUSE vs. PP2A‐KD‐CUSE) and the number of sniffing of the novel object (Figure 7F: Kruskal–Wallis statistic = 25.23, *p* < 0.0001). In addition, hippocampal PP2A‐KD abolished the beneficial effects of voluntary running on restoring spatial memory in Y maze (Figure 7G: Kruskal–Wallis statistic = 34.99, *p* < 0.0001 & Figure 7H: Kruskal–Wallis statistic = 22.33, *p* < 0.0001), and on reducing anxiety‐like behavior in OFT (Figure 7I: Kruskal–Wallis statistic = 17.51, *p* = 0.0006) in stressed mice. There was no significant difference in locomotor activity among groups (Figure 7J: *F*_3_,_59_ = 2.367, *p* = 0.8841, CUSE vs. PP2A‐KD‐CUSE). Collectively, these results indicate that hippocampal PP2A activity is required for running to reduce Tau hyperphosphorylation and improve behavioral deficits in stressed mice.

### 3.8 PP2A Is Required for Physical Exercise to Restore Adult Hippocampal Neurogenesis

**FIGURE 8:**
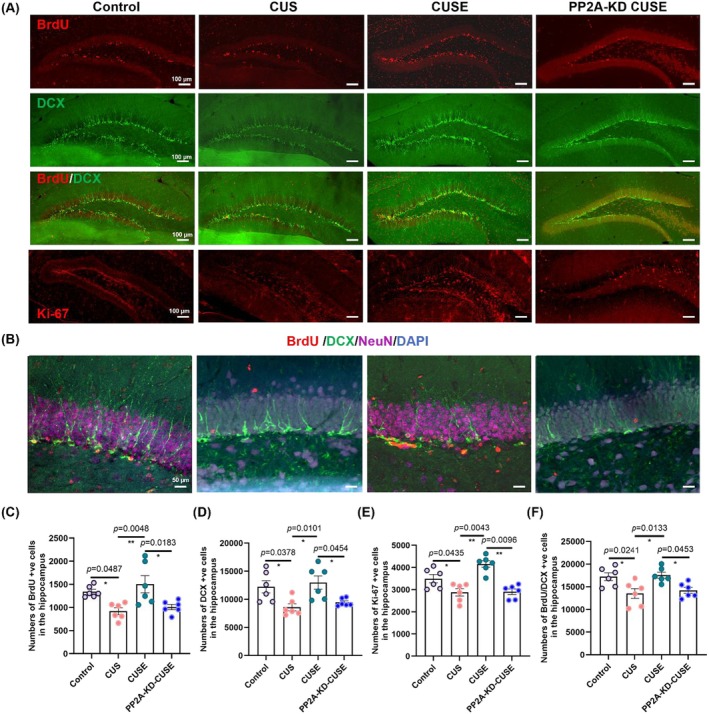
PP2A knockdown abolished the rescuing effects of running on adult hippocampal neurogenesis and synaptic protein expression in stressed mice. (A) Representative immunofluorescent images of hippocampal dentate gyrus. Scale bar, 100 μm. (B) The representative co‐labeling of BrdU^+^/DCX^+^/NeuN^+^ newborn neurons in the hippocampus. Scale bar, 50 μm. (C) Quantification of surviving cells, (D) proliferating cells, and (E) immature neurons in the hippocampus. (F) Quantification of the number of BrdU^+^/DCX^+^ newborn neurons in the hippocampus. *p < 0.05, **p < 0.01; *N* = 48–54 slices from 6 mice per genotype. Data were analyzed using one‐way analysis of variance followed by Tukey's post hoc test for intergroup comparison and the Kruskal–Wallis test with Dunn's multiple comparisons for multiple‐group comparisons. All data are presented as mean ± SEM.

Immunostaining revealed a significant reduction in the number of surviving newborn cells in stressed mice with and without PP2A expression compared to non‐stressed runners (Figure 8C, BrdU: *F*_3_,_20_ = 6.669, *p* = 0.0027 and Figure 8A). PP2A‐KD reduced the number of proliferating cells and immature neurons and diminished the effects of running on adult neurogenesis (Figure 8D, DCX: *F*_3_,_20_ = 5.855, *p* = 0.0049; Figure 8E, Ki‐67: *F*_3_,_20_ = 6.827, *p* = 0.0024; Figure 8A), and PSD‐95 protein expression in stressed mice (Figure 8E: *F*_3_,_20_ = 12.08, *p* < 0.0001). Taken together, these findings indicate the crucial role of hippocampal PP2A in mediating the beneficial effects of voluntary running on restoring adult neurogenesis, Tau phosphorylation and cognitive performance.

## 4 Discussion

Our findings identify the adiponectin–PP2A pathway as a critical molecular mechanism underlying the effects of physical exercise in lowering Tau hyperphosphorylation and restoring hippocampal plasticity under chronic stress condition. Using gain‐ and loss‐of‐function approaches, we demonstrate that both adiponectin and hippocampal PP2A are required for exercise to reduce Tau hyperphosphorylation, restore adult neurogenesis, and rescue cognitive deficits.

By using a mouse model with CUS, we investigated whether physical exercise in terms of voluntary running increases adiponectin levels to modulate PP2A activity, which in turn reduces Tau hyperphosphorylation and enhances hippocampal structural plasticity. Chronic stress is known to elevate corticosterone levels and promote Tau hyperphosphorylation (Ochi et al. 2024; Wu et al. 2018), we therefore adopted this model to examine stress‐associated AD‐like pathology (Burke et al. 2024). We found that chronic stress increased Tau hyperphosphorylation in the hippocampus (Figure 2C–I), consistent with the results of other studies indicating that chronic stress could be a risk factor for the development of AD (Denisenko et al. 2023; Graham et al. 2022; Saeedi and Rashidy‐Pour 2021). This model enables us to test the underlying mechanisms of physical exercise‐reduced Tau phosphorylation.

Clinical studies have shown that physical exercise is effective in preventing AD (Hoffmann et al. 2016; D. Li et al. 2024; López‐Ortiz et al. 2021). Our behavioral studies demonstrated that physical exercise effectively reversed cognitive and behavioral deficits induced by chronic stress (Figure 1), consistent with our previous research (Yau et al. 2014; W. Zhang et al. 2021) and studies by others (Ghalandari‐Shamami et al. 2021; D. M. Kim and Leem 2016). Notably, our exercise regimen consisted of three weeks of voluntary wheel running in stressed mice, which may differ from protocols in prior studies that reported anxiolytic effects using forced treadmill running (Yan et al. 2022), or longer duration with voluntary running (Kodali et al. 2021), or different age groups (Liśkiewicz et al. 2020) in different animal models (Wu et al. 2020). Additionally, the timing of behavioral testing relative to the last exercise session may influence results. In our study, OFT was conducted 24 h after the last exercise session, as shown in Figure 1A, whereas some studies performed tests immediately after exercise. It is also possible that the anxiolytic effects of exercise are more readily detected in other behavioral paradigms (e.g., elevated plus maze) or under conditions of heightened baseline anxiety, which may not be present in our wildtype mice. Additionally, the current study focuses on a short‐term effect with a 3‐week exercise/stress paradigm, a longer‐term timepoint (e.g., 8–12 weeks) to assess sustained effects on Tau pathology warrants future study.

Tau hyperphosphorylation is a central pathological feature of AD (Ye et al. 2024) and contributes directly to synaptic dysfunction (Sohn et al. 2019) and memory impairment (Richetin et al. 2020). From clinical diagnosis to targeted therapy, Tau protein represents a promising research direction for understanding and treating AD and other tauopathies (Creekmore et al. 2024). While physical exercise has been widely reported to improve cognitive performance (Choi et al. 2024) and reduce β‐amyloid (Ferrarelli 2023) and Tau pathology (Abdullahi et al. 2024), the molecular pathways linking peripheral metabolic adaptation to central Tau regulation remain incompletely understood. Our findings provide evidence that exercise attenuates stress‐induced Tau hyperphosphorylation through an adiponectin‐dependent activation of PP2A, thereby connecting adiponectin to Tau homeostasis in the hippocampus.

### 4.1 Adiponectin Is a Critical Mediator for Physical Exercise to Improve Hippocampal Function

Our data identify adiponectin as an essential mediator for physical exercise to reduce Tau hyperphosphorylation. Adiponectin deficiency abolished the beneficial effects of physical exercise in improving memory performance, restoring adult hippocampal neurogenesis, enhancing synaptic plasticity, and reducing p‐Tau levels (Figure 4). Previous studies have demonstrated that adiponectin/AdipoR1 signaling critically mediates the antidepressant effects of voluntary running by enhancing adult hippocampal neurogenesis (Yau et al. 2014). While activating adiponectin receptors via the agonist AdipoRon mimics the effects of exercise on promoting adult neurogenesis and synaptic plasticity, resulting in hippocampal‐dependent learning and memory improvement (Formolo et al. 2019; Lee et al. 2021). Together, these convergent findings position adiponectin as a critical factor regulating hippocampal structural and functional remodeling.

There is a lack of evidence showing a significant correlation between serum and hippocampal adiponectin levels. We have previously demonstrated a significant negative correlation between adiponectin levels in the prefrontal cortex and immobility time in the exercised mice (Cheng et al. 2025). Higher hippocampal adult neurogenesis is correlated with higher adiponectin levels (Yau et al. 2014), whereas lower blood adiponectin levels are correlated with depression severity in patients (Diniz et al. 2012). Adiponectin is predominantly produced by adipocytes and is the most abundant plasma protein; however, its concentration in cerebrospinal fluid is approximately 1000‐fold lower than in blood (Kos et al. 2007). Only the low molecular weight forms of adiponectin, such as the trimeric form, can cross the blood–brain barrier (BBB) (Kubota et al. 2007; Schön et al. 2019), and this transport may be influenced by BBB integrity and local receptor expression. Therefore, the link between hippocampal and serum adiponectin levels requires further studies to be elaborated in the context of the central nervous system.

Although adiponectin is primarily secreted by adipocytes, both AdipoR1 and AdipoR2 are expressed in the brain (Yau et al. 2014). Given the relatively low levels of adiponectin detected in the brain compared to the circulation, its effects are likely mediated through its receptor rather than substantial local synthesis in the brain. Immunohistochemical and in situ hybridization analyses have demonstrated that AdipoR1 and AdipoR2 are widely distributed in neurons throughout the hippocampus, cortex, and other brain regions, and are also present in astrocytes (Qi et al. 2004; Thundyil et al. 2012). There is limited evidence for adiponectin expression in microglia, and most of the neurobiological effects of adiponectin are thought to be mediated through its receptors on neurons and astrocytes (Ng et al. 2016).

Consistent with the role of chronic stress in accelerating brain aging and Tau phosphorylation (Ochi et al. 2024; Wu et al. 2018), we reported that CUS increased hippocampal p‐Tau, whereas voluntary running showed the opposite effect in an adiponectin‐dependent manner. Adiponectin elicits pro‐cognitive effects (Ng et al. 2021; Yau et al. 2014) and neuroprotective effects against β‐amyloid‐induced neurotoxicity (Jian et al. 2019) similar to those of physical exercise. Furthermore, adiponectin improves memory performance by attenuating Tau phosphorylation and Aβ accumulation in *Adipo* ^−/−^ mice (Ng et al. 2016). Consistently, our findings showed that mice with adiponectin deficiency displayed memory impairments (Figure 3A–E) and that the deficiency abolished the beneficial effects of voluntary running on hippocampal plasticity (Figure 5), suggesting the critical role of adiponectin in mediating the neuroprotective effects of physical exercise. It is known that adiponectin regulates synaptic plasticity (Thacker et al. 2024), and neuroinflammation (Jian et al. 2019; L. Liu et al. 2024), adult neurogenesis (Yau et al. 2014). All of these could be affected by adiponectin deficiency, leading to impaired learning and memory (Ng et al. 2016). Therefore, decrease in adiponectin could reduce hippocampal structural and synaptic plasticity, resulting in hippocampal impairment, hence cognitive impairment as observed in *Adipo* ^−/−^ mice.

Although few clinical studies have reported a higher level of circulatory adiponectin in patients with mild cognitive impairment (MCI) or AD, when compared to cognitively normal controls (Horgusluoglu et al. 2022; J. W. Kim et al. 2023; Une et al. 2011), one study reported that a higher blood level of adiponectin is associated with lower ventricle volume (Garcia‐Garcia et al. 2024). Since adiponectin exists in different isoforms and only the trimeric form of adiponectin can pass through the BBB, further investigation is warranted to determine if this is the compensatory mechanism for reduction of adiponectin levels in the brain.

In contrast, PP2A dysregulation presents a more consistent pattern across human studies. PP2A dysfunction triggers activation of the classical complement cascade, leading to increased Tau phosphorylation (Jun et al. 2022). Significant reductions in PP2A mRNA (Vogelsberg‐Ragaglia et al. 2001), PP2A protein (Guadagna et al. 2012; Jun et al. 2022; Zhao et al. 2003), and its activity (Shentu et al. 2018) in the brain tissue of AD and MCI patients underscores its role in AD pathogenesis. Given that PP2A accounts for the majority of Tau dephosphorylating activity in neurons, its decline is strongly implicated in pathological Tau accumulation. Together, these findings suggest that impaired adiponectin levels and reduced PP2A activity could contribute to Tau hyperphosphorylation.

### 4.2 Physical Exercise Increased PP2A Activity to Reduce p‐Tau and Improve Hippocampal Neuroplasticity in an Adiponectin‐Dependent Manner

Our hippocampal‐specific PP2A knockdown confirmed that PP2A activity is necessary for physical exercise to improve memory performance in stressed mice. The absence of hippocampal PP2A expression abolished the effects of physical exercise on reducing Tau hyperphosphorylation (Figure 7B–D), restoring hippocampal adult neurogenesis (Figure 8A–D) and PSD‐95 levels (Figure 8E) in stressed mice, suggesting that PP2A is critical for physical exercise to protect the hippocampus against chronic stress‐induced p‐Tau accumulation and impairments in neuroplasticity. These findings echo prior observations that exercise enhances PP2A activity under stress conditions (W. Zhang et al. 2021) and provide mechanistic evidence that adiponectin is required for PP2A activation.

PP2A is known to be a Tau dephosphorylating phosphatase (W. Hu et al. 2022; Z. Hu et al. 2023; R. Liu et al. 2008). It contributes to nearly 70% of Tau dephosphorylating activity in neurons, making it a promising target for reducing Tau hyperphosphorylation in AD (Clark and Ohlmeyer 2019). Physical exercise reduces Tau protein phosphorylation levels and enhances PP2A activity in stressed mice (W. Zhang et al. 2021). In the present study, our data revealed that voluntary running increases PP2A activity in the hippocampus and consequently alleviates Tau hyperphosphorylation. PP2A methylation and holoenzyme activity can impact Tau phosphorylation and amyloid precursor protein processing (Rasool et al. 2023; Sontag et al. 2007). LCMT1 is indispensable in modulating PP2A methylation, which is important for the cell cycle and cell survival (Stanevich et al. 2011). Conversely, PME‐1, a key phosphatase involved in Tau dephosphorylation, is an endogenous inhibitor that negatively regulates PP2A (Sents et al. 2013; Staniszewski et al. 2020). PME‐1 and LCMT1 work in concert to control PP2A activity, and thus regulate Tau (de)phosphorylation (Martin et al. 2013). Our results revealed that adiponectin knockout significantly decreased PP2A activity in the hippocampus (Figure 4B and Figure 6B) and increased LCMT1 expression (Figure 6D,H). Our data indicate that adiponectin deficiency disrupts PP2A activity and alters components of this regulatory machinery, which suggests that adiponectin modulates PP2A methylation to influence Tau phosphorylation (Figure 6F,H).

Our data has demonstrated that PP2A activity was reduced in stressed mice, and this decrease can be rescued by physical exercise. PP2A holoenzyme is a heterotrimeric complex, consisting of a core dimer of a catalytic C subunit (PP2Ac) and a scaffolding A subunit (PR65), along with one regulatory B subunit (Neale et al. 2025). Changes in the activity of PR65 and PP2A B55α subunit have not been investigated in the current work. Thus, assessing PP2A holoenzyme subunits could provide further mechanistic insights. Additionally, previous studies have demonstrated that pharmacological activation of PP2A by FTY720 administration can reduce Tau phosphorylation. Future studies incorporating a PP2A activator as a positive control would be valuable to confirm the critical role of this enzyme in reducing Tau hyperphosphorylation. Furthermore, such experiments could determine if PP2A activation alone is sufficient to mimic the beneficial effects of physical exercise on hippocampal function.

It should be noted that our study does not directly test whether PP2A activation alone, such as through pharmacological agonists, is sufficient to mimic the beneficial effects of exercise. Therefore, it remains unclear whether pharmacological activation of PP2A could mimic the effects of physical exercise. Future studies employing specific PP2A agonists or genetic approaches will be necessary to determine whether PP2A activation alone can recapitulate the outcomes associated with exercise. As female hormones such as estrogen and follicle‐stimulating hormone can significantly affect cognition (Russell et al. 2019; Xiong et al. 2022), to exclude the possible influence of female hormones, male mice were used in this study. However, females exhibit a higher risk of AD and often more severe cognitive decline (Vila‐Castelar et al. 2023). Notably, females have higher levels of adiponectin than males (Combs et al. 2003; Nishizawa et al. 2002). Both adiponectin levels and PP2A activity may be modulated by sex hormones, and their neuroprotective effects could differ between males and females. Therefore, it remains unclear whether the mechanisms we identified in male mice are equally applicable to female mice. Future investigations including both sexes will be essential to determine whether the effects of adiponectin and PP2A on regulating hippocampal function and Tau pathology are sex dependent.

Emerging evidence suggests potential involvement of GSK‐3β signaling in this adiponectin‐PP2A axis regulatory Tau phosphorylation. The PI3K‐GSK‐3β signaling controls the production of adiponectin to regulate metabolism (Chen et al. 2016). Notably, the Wnt/GSK‐3β/β‐catenin pathway activates PPARγ, thereby mediating adipogenesis (Okamura et al. 2014). AdipoR1 activation has been linked to modulation of GSK‐3β phosphorylation (Guo et al. 2015). GSK‐3β inhibits the demethylation of PP2A at leucine‐309 through regulation of LCMT1 and PME‐1 (Yao et al. 2012). Consistently adiponectin deficiency reduced GSK‐3β phosphorylation in parallel with diminished PP2A activity (Figure 6G,H). However, it remains unclear whether adiponectin interacts with GSK‐3β to regulate PP2A activity. Further studies are needed to determine whether inhibition of GSK‐3β (e.g., with lithium) can restore PP2A activity or reduce Tau phosphorylation in *Adipo* ^−/−^ mice. Beyond GSK‐3β, PP2A may interact with other pathways. The PI3K‐Akt pathway exhibits bidirectional crosstalk with PP2A, as evidenced by their cooperative control of Tau phosphorylation dynamics (Y. Wang et al. 2015). PP2A demonstrates context‐dependent modulation of Akt signaling in small T cell environments (Andrabi et al. 2007). Furthermore, PP2A negatively regulates AMPK activity (Park et al. 2013). Notably, our previous data reveal that exercise‐induced activation of AMPK in the hippocampus was compromised in *Adipo* ^−/−^ mice (Yau et al. 2014). Previous study suggested that adiponectin‐activated AMPK initiates a phosphatase cascade through PP2A activation, ultimately leading to AKT dephosphorylation (K. Y. Kim et al. 2009). This integrated mechanism positions PP2A as a critical signaling that fine‐tunes both AMPK and Akt pathways, however, the detailed regulatory network linking adiponectin signaling and PP2A interaction warrants further investigation. Furthermore, while our current study focuses on adult neurogenesis and PSD‐95 levels, the long‐term potentiation and long‐term depression were not investigated in our PP2A‐KD model. Since changes of hippocampal plasticity also include dendritic and spine remodeling, as well as synaptic plasticity, future study should also examine other forms of hippocampal plasticity including long‐term potentiation/long‐term depression, dendritic remodeling and spine density in addition to adult hippocampal neurogenesis.

## 5 Conclusion

In summary, chronic stress exposure increases p‐Tau phosphorylation and reduces adiponectin levels in the hippocampus, suggesting that chronic stress exposure could be a risk factor for increased p‐Tau accumulation in the brain, thereby increasing the risk for developing AD. Conversely, voluntary running exerts significant neuroprotective effects by preventing cognitive impairment and Tau hyperphosphorylation in the hippocampus. Our results suggest a novel mechanism by which physical exercise increases adiponectin to increase PP2A activity, leading to reduced hippocampal p‐Tau levels. These results not only provide mechanistic insights into the effects of physical exercise on preventing p‐Tau accumulation in the hippocampus, but also suggest that increasing adiponectin and PP2A activity could be potential therapeutic strategies for reducing Tau hyperphosphorylation.

## Author Contributions

Conceptualization: Hui‐Hui Guo, Suk‐Yu Yau, Hai‐Ning Ou, Hector Wing‐Hong Tsang. Methodology: Hui‐Hui Guo, Suk‐Yu Yau, Jia‐Sui Yu. Investigation and data analysis: Hui‐Hui Guo, Jia‐Sui Yu, Suk‐Yu Yau. Writing preparation: Hui‐Hui Guo, Hai‐Ning Ou, Jia‐Sui Yu, Zi‐Rui Luo, Suk‐Yu Yau, Hector Wing‐Hong Tsang. Funding acquisition: Suk‐Yu Yau, Hai‐Ning Ou, Hector Wing‐Hong Tsang. Research resources: Hai‐Ning Ou, Zi‐Rui Luo, Suk‐Yu Yau, Hector Wing‐Hong Tsang.

## Funding

This project was supported by funding from National Natural Science Foundation of China (General Program 82072529), Key Laboratory of Guangdong Higher Education Institutes (2021KSYS009), the China Postdoctoral Science Foundation (Grant 2022M720907) and seed funding supported by Mental Health Research Center (MHRC) at Hong Kong Polytechnic University.

## Ethics Statement

All animal experiments were performed according to the Institutional Animal Care and Use Committee (IACUC) Guidelines on the Use of Laboratory Animals and were approved by the Hong Kong Polytechnic University (PolyU) Research Committee on Animal Care (ASESC Case No.: 20‐21/303‐RS‐R‐NSFC), and performed in compliance with the National Institutes of Health Guide for the Care and Use of Laboratory Animals. No human study is involved.

## Consent

The authors have nothing to report.

## Conflicts of Interest

The authors declare no conflicts of interest.

## Supporting information


**Data S1:** acel70447‐sup‐0001‐supinfo.docx.

## Data Availability

All data associated with this study are present in the paper and the Supporting Information. Raw datasets will be made available by the authors on request.
