# Supplementary material for: The Adiponectin‐PP2A Pathway Confers Cognitive Benefits of Physical Exercise Against Chronic Stress‐Induced Tau Hyperphosphorylation in the Hippocampus
Source: Aging Cell. 2026 Mar 17;25(3):e70447. doi: 10.1111/acel.70447 (PMC13093838; doi:10.1111/acel.70447)
Supplement: Supplementary file 1 — Data S1: acel70447‐sup‐0001‐supinfo.docx. [file ACEL-25-e70447-s001.docx]

**Supplementary materials**

**Supplementary figures**

**
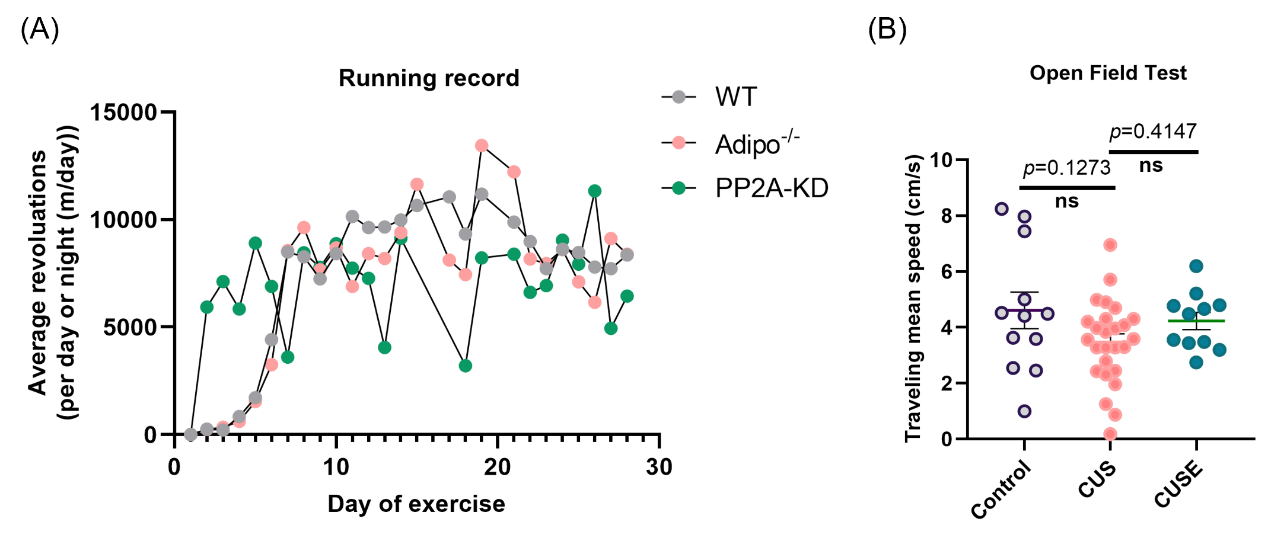
**

**Figure S1. Physical exercise did not affect locomotor activity**

(A) Average daily voluntary running distance (m/day) converted by wheel revolutions. Data points represent the mean daily distance per wheel for each genotype. (B) No difference in travel mean speed among the groups. “ns” means non-significant difference. Data were analyzed using by One-way analysis of variance followed by Tukey’s post hoc test for intergroup comparison and the Kruskal-Wallis test with Dunn's multiple comparisons for multiple-group comparisons. All data were presented as mean ± SEM.

**
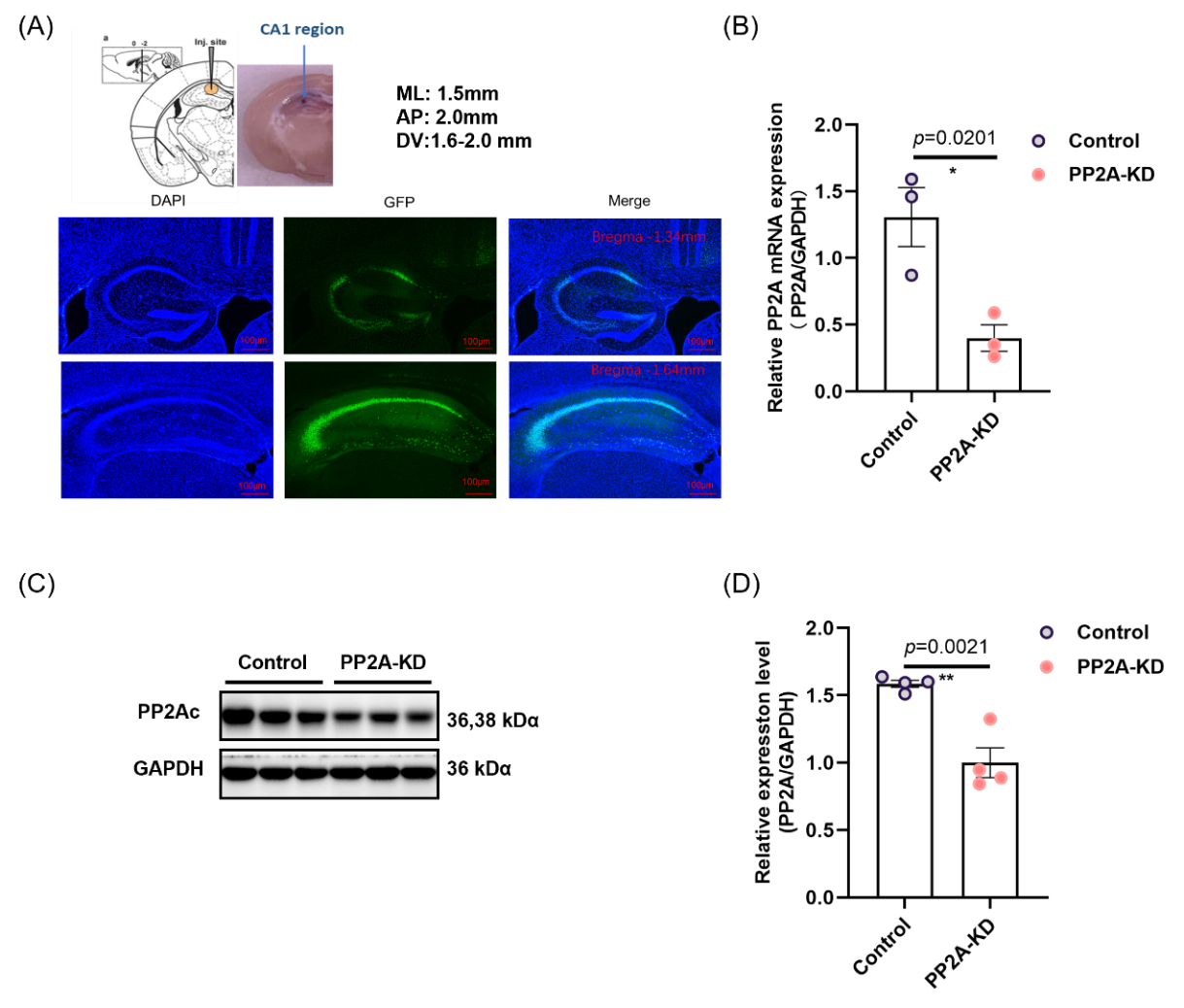
**

**Figure S2. Validation of hippocampal-specific PP2A knockdown using AAVs in combination with PP2a-flox mice.**

(A) AAV injection targeting hippocampal CA1 region with GFP expression. (B) Successful knockdown of PP2A (PP2A-KD) with decreased mRNA expression level in the hippocampus. **p* < 0.05; N=3. (C) Representative western blotting images. (D) PP2A protein quantifications using Western blotting. ***p* < 0.001; N = 4 mice. Data were analyzed by the student’s t-test or Kolmogorov-Smirnov test for two-group comparisons. All data were presented as mean ± SEM.


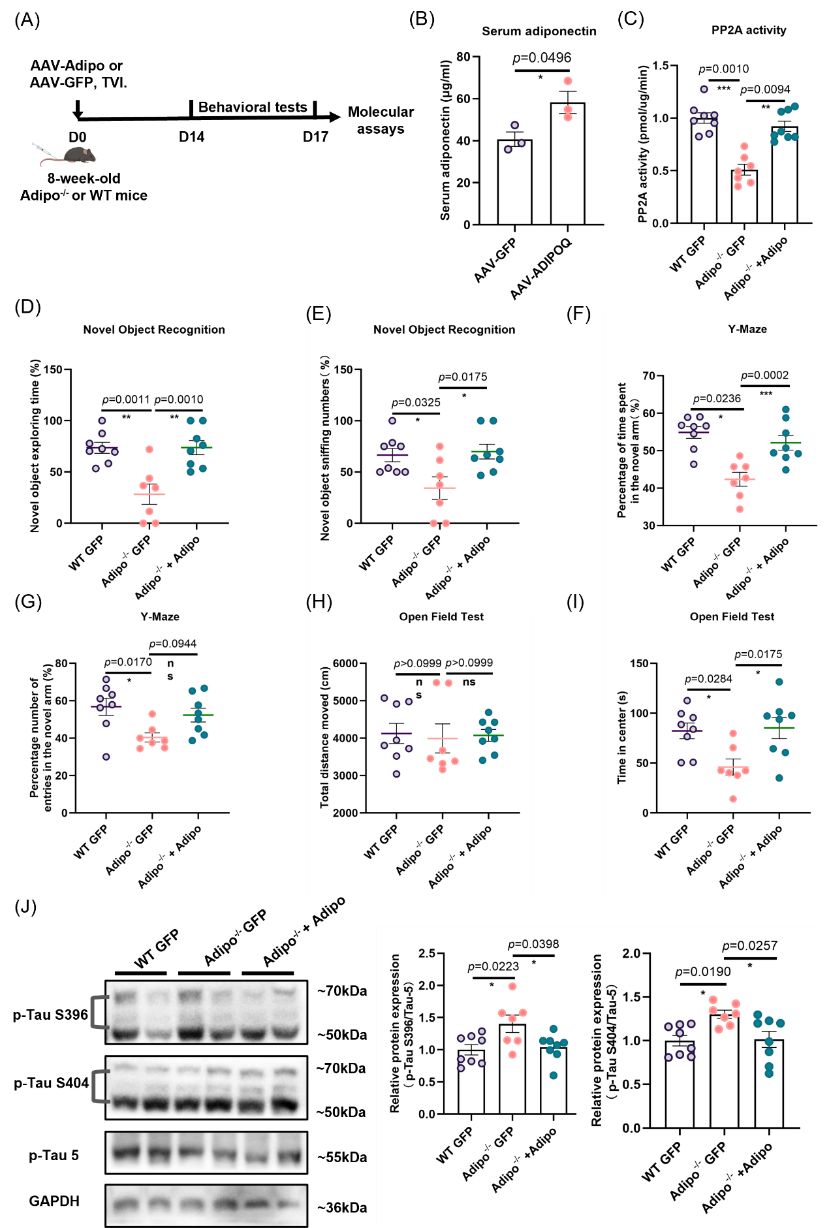


**Figure S3. Increased adiponectin enhances the PP2A activity and rescues cognitive impairment in *Adipo^-/-^* mice.**

(A) Experiment timeline with 2-week AAV supplementation in WT and *Adipo^-/-^* mice paradigm, followed by a battery of behavioral tests or molecular assays. (B) Overexpression of adiponectin via AAV increased serum apelin levels. *p < 0.05; N = 3 per group in control and AAV-adipo. (C) PP2A activity assay. (D) Percentage of time spent and (E) numbers of sniffing to the novel object in the NOR test. **p* < 0.05, ***p* < 0.01. (F) Percentage of time spent and (G) number of visits to the novel arm in the Y-maze. **p* < 0.05, ****p* < 0.001. (H) The total travelling distance in the open field test, and (I) the total time spent in the central area, **p* < 0.05. (J) Western blotting analysis of p-Tau S396 and p-Tau S404 proteins in the hippocampus. *p < 0.05. Data were analyzed using by One-way analysis of variance followed by Tukey’s post hoc test for intergroup comparison and the Kruskal-Wallis test with Dunn's multiple comparisons for multiple-group comparisons. N = 8, 7 and 8 mice in WT GFP, *Adipo^-/-^* GFP, and *Adipo^-/-^* +Adipo groups, respectively. All data are presented as mean ± standard error of means (SEM).


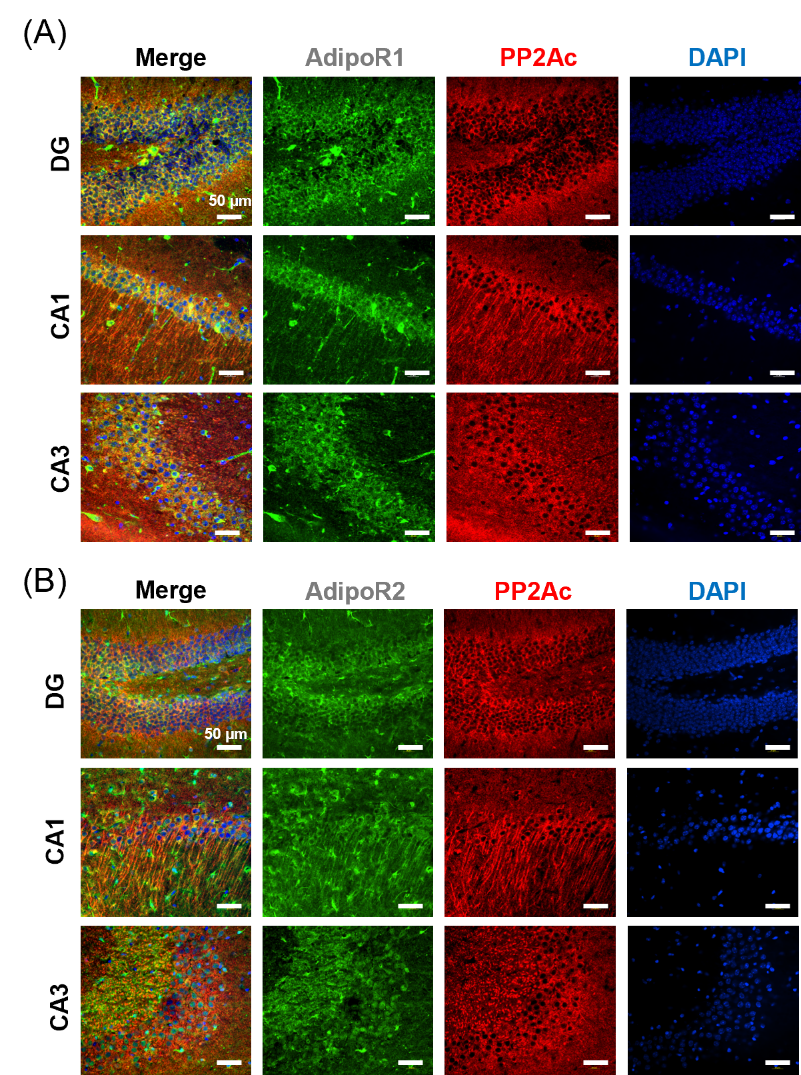


**Figure S4. Immunofluorescent co-labeling of PP2A with AdipoR1 or AdipoR2 in the hippocampal neurons.**

(A) Co-labeling of PP2A with AdipoR1, and (B) co-labeling of PP2A with AdipoR2 in the hippocampal dentate gyrus (DG), Cornu Ammonis 1 (CA1), and CA3.

**Supplementary tables**

**Table S1 Chronic unpredictable stress protocol**

| **Day** | **Stressors** | **Day** | **Stressors** |
| --- | --- | --- | --- |
| 1 | Restraint stress (0.5h) | 12 | Empty bottle exposure (1h) |
| 2 | Inescapable foot shock (5 min – 5mA) | 13 | Wet bedding (maximum 24h) |
| 3 | Exposure to the shock apparatus (without shock- 1h) | 14 | Paired housing (1h) |
| 4 | Paired housing (1h) | 15 | Inescapable foot shock (5 min – 5mA) |
| 5 | Cage tilt (maximum 24h) | 16 | Exposure to the shock apparatus  (without shock 1h) |
| 6 | Restraint stress (1.5h) | 17 | Wet bedding (maximum 24h) |
| 7 | Paired housing (1h) | 18 | Food and water deprivation  (maximum 24h) |
| 8 | Wet bedding (maximum 24h) | 19 | Empty bottle exposure (1h) |
| 9 | Paired housing (1h) | 20 | Cage tilt (maximum 24h) |
| 10 | Restraint stress (2h) | 21 | Restraint stress (2.5h) |
| 11 | Food and water deprivation  (maximum 24h) |  |  |

**Table S2 List of primers for RT-qPCR**

| **Gene Name** | **Primer (**5’ to 3’**)** |
| --- | --- |
| PP2A-F | ATGGA CGAGA AGTTG TTCAC C |
| PP2A-R | CAGTG ACTGG ACATC GAACC T |
| GAPDH-F | TTCCTACCCCCAATGTATCCG |
| GAPDH-R | CATGAGGTCCACCACCCTGTT |
